# Natural combinatorial genetics and prolific polyamine production enable siderophore diversification in *Serratia plymuthica*

**DOI:** 10.1186/s12915-021-00971-z

**Published:** 2021-03-15

**Authors:** Sara Cleto, Kristina Haslinger, Kristala L. J. Prather, Timothy K. Lu

**Affiliations:** 1 Department of Electrical Engineering and Computer Science, Massachusetts Institute of Technology, Cambridge, MA USA; 2 Department of Biological Engineering, Massachusetts Institute of Technology, Cambridge, MA USA; 3 Synthetic Biology Center, Massachusetts Institute of Technology, Cambridge, MA USA; 4 Department of Chemical Engineering, Massachusetts Institute of Technology, Cambridge, MA USA; 5 Department of Chemical and Pharmaceutical Biology, University of Groningen, Groningen, The Netherlands

**Keywords:** Natural products, Siderophores, Pathway elucidation, Polyamines

## Abstract

**Background:**

Iron is essential for bacterial survival. Bacterial siderophores are small molecules with unmatched capacity to scavenge iron from proteins and the extracellular milieu, where it mostly occurs as insoluble Fe^3+^. Siderophores chelate Fe^3+^ for uptake into the cell, where it is reduced to soluble Fe^2+^. Siderophores are key molecules in low soluble iron conditions. The ability of bacteria to synthesize proprietary siderophores may have increased bacterial evolutionary fitness; one way that bacteria diversify siderophore structure is by incorporating different polyamine backbones while maintaining the catechol moieties.

**Results:**

We report that *Serratia plymuthica* V4 produces a variety of siderophores, which we term the *siderome*, and which are assembled by the concerted action of enzymes encoded in two independent gene clusters. Besides assembling serratiochelin A and B with diaminopropane, *S. plymuthica* utilizes putrescine and the same set of enzymes to assemble photobactin, a siderophore found in the bacterium *Photorhabdus luminescens*. The enzymes encoded by one of the gene clusters can independently assemble enterobactin. A third, independent operon is responsible for biosynthesis of the hydroxamate siderophore aerobactin, initially described in *Enterobacter aerogenes*. Mutant strains not synthesizing polyamine-siderophores significantly increased enterobactin production levels, though lack of enterobactin did not impact the production of serratiochelins. Knocking out SchF0, an enzyme involved in the assembly of enterobactin alone, significantly reduced bacterial fitness.

**Conclusions:**

This study shows the natural occurrence of serratiochelins, photobactin, enterobactin, and aerobactin in a single bacterial species and illuminates the interplay between siderophore biosynthetic pathways and polyamine production, indicating routes of molecular diversification. Given its natural yields of diaminopropane (97.75 μmol/g DW) and putrescine (30.83 μmol/g DW), *S. plymuthica* can be exploited for the industrial production of these compounds.

**Supplementary Information:**

The online version contains supplementary material available at 10.1186/s12915-021-00971-z.

## Background

Iron, one of the most abundant elements on Earth [1], is crucial for the survival of all living organisms, including bacteria. It occurs in two forms: soluble (Fe^2+^) and insoluble (Fe^3+^). Soluble iron can be readily taken up by aerobic microorganisms (but not anaerobes), although it is uncommon at pH 7 [2–4]. Bacteria and most life forms have evolved a diversity of ways that converge to the same goal: obtaining soluble iron (Fe^2+^) for survival. They have devised complex regulatory mechanisms responding to Fe^2+^ unavailability that induce the expression of a series of genes to produce small iron chelators, termed siderophores [5–7], secrete them, and take up their iron-bound forms. Bacteria have not only devised ways of biosynthesizing “proprietary” siderophore molecules, but have evolved transport mechanisms that allow them to utilize foreign siderophores, or xenosiderophores, as well [8, 9]. This mechanism has led siderophores to be considered public goods, traded between bacteria and impacting their survival [10–14]. Some bacteria have evolved extraordinary ways to synthesize proprietary siderophores that require the expression of specialized TonB-dependent receptors (TBDRs) to allow for efficient siderophore uptake by the producer and its relatives [15]. One such innovative way is the incorporation of polyamines into the nascent siderophore, which has evolved in multiple species that naturally produce polyamines. Thus, diaminopropane (DAP) is incorporated into serratiochelins in *Serratia plymuthica* [16], norspermidine is incorporated into vibriobactin in *Vibrio cholerae* [17] and vulnibactin in *Vibrio vulnificus* [18], putrescine is incorporated into photobactin in *Photorhabdus luminescens* [19], and spermidine is incorporated into parabactin in *Paracoccus denitrificans* [20] and agrobactin in *Agrobacterium tumefaciens* [21].

Polyamines are small organic molecules with various numbers of carbons and amine moieties and a flexible structure [22, 23]. They are synthesized by most bacteria [24] and eukaryotes [25] from l-lysine, l-methionine, l-aspartate, and l-arginine, with bacteria synthesizing a greater diversity of polyamines than eukaryotes. These moieties are incorporated into the nascent siderophore molecules by dedicated amide synthases, which contain stand-alone condensation domains structurally related to those found in non-ribosomal peptide synthetases [26, 27]. Amide synthases have already been identified in several organisms that produce polyamine-containing siderophores, such as PhbG in *Photorhabdus* spp. [19], SchH in *Serratia* spp. [16], and especially VibH in *Vibrio* spp. [27]. VibH has been crystalized and the condensing activity thoroughly studied [27]. The amide synthase involved in the assembly of agrobactin in *Agrobacterium* spp. has yet to be identified though its biosynthetic cluster is known [28]. The biosynthetic cluster for parabactin, thus also its amide synthase, has yet to be identified.

*S. plymuthica* stands out for its ability to produce the nonribosomal peptide antibiotic zeamine and the nonribosomal peptide siderophores, the serratiochelins [29, 30]. Gene clusters evolutionarily obtained by *S. plymuthica* from a diversity of bacteria, such as *Dickeya zeae* [29, 30], *Escherichia coli*, and *Vibrio* spp. [16], are involved in the assembly of these molecules. In this work, we further explored and elucidated the diversity of siderophores produced by *S. plymuthica* and dissected the interplay of two catechol siderophore pathways with a superpathway (i.e., a network of closely related and interconnected biosynthetic pathways) for polyamine production, as well as their role in the diversification of catechol siderophores in this organism. In addition, to shed light on the relationship between the amide synthases and their preference for specific polyamines, we identified active site residues using bioinformatics tools. Furthermore, we dissected the diversity of putative TBDRs in the genome of *S. plymuthica*.

In this work, *S. plymuthica* was found to produce an extraordinary diversity of siderophores, which we termed the *siderome*. This diversity is generated by an interplay of three independent siderophore biosynthetic clusters and a prolific polyamine production superpathway, which is rare among Enterobacteriaceae. These siderophores were serratiochelin A and B, enterobactin, photobactin, and aerobactin. To the best of our knowledge, this is the first published natural occurrence of serratiochelins, photobactin, enterobactin, and aerobactin in a single bacterial species. These findings suggest that the capacity of *S. plymuthica* to accrue biosynthetic clusters that evolved in other organisms is more extensive than so far described. Our results emphasize the utility of studying the evolution of natural product biosynthetic pathways and networks.

## Results

### Characterization of the siderophores produced by *S. plymuthica*

**Fig. 1:**
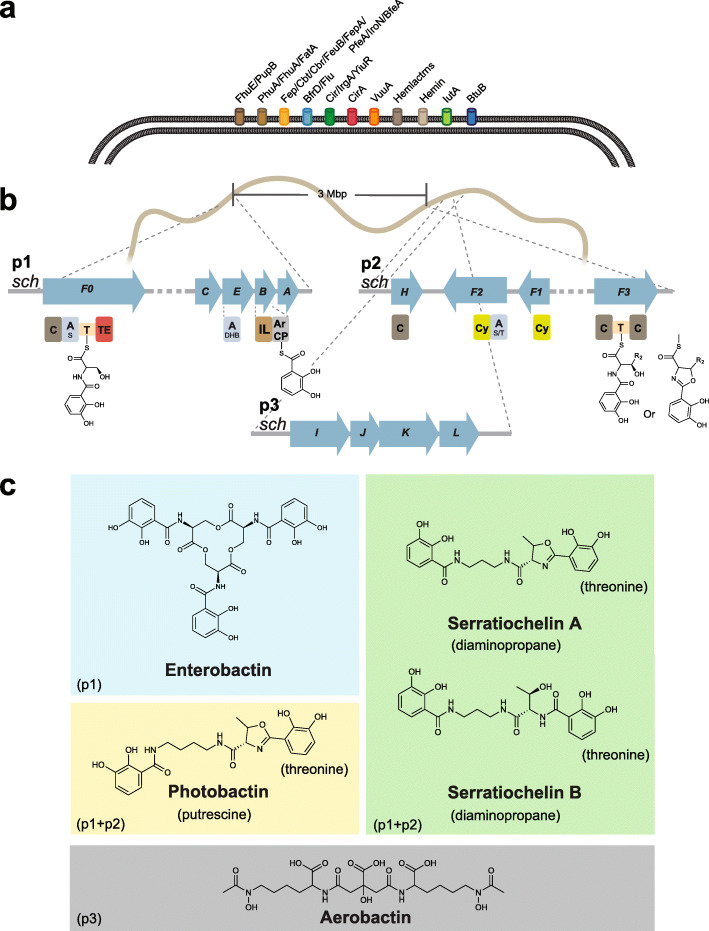
Schematic of the molecular players in the iron uptake mechanism of *S. plymuthica*. Putative TonB-dependent siderophore uptake receptors identified in the *S. plymuthica* genome (**a**); siderophore-encoding gene clusters (p1–p3) identified in the *S. plymuthica* genome and experimentally characterized in this study (domain annotations below blue arrow: C = condensation domain, A = adenylation domain (index indicates the substrate; 2,3-dihydroxybenzoate (DHB), serine (S), threonine (T)), T = thiolation domain, IL = isochorismate lyase, ArCP = aryl carrier protein, TE = thioesterase domain, Cy = cyclisation domain) (**b**); siderophores detected in this study grouped by their underlying biosynthetic pathways (p1–p3) (**c**)

In order to assess the capability of *S. plymuthica* V4 to produce siderophores, we analyzed culture supernatant extracts using liquid chromatography coupled tandem mass spectrometry. We analyzed the fragmentation pattern of each compound by comparing it to predicted or known patterns of siderophores from other organisms. We determined that, alongside the signature siderophores serratiochelin A and B [16], *S. plymuthica* produces enterobactin, photobactin, and aerobactin known from other organisms [31, 32] [19] [33] (Fig. 1 and Additional file 1: Additional Figs. S1 through S4). Given this observation, we next sought to investigate the biosynthetic pathways that give rise to these well-known siderophores in *S. plymuthica* V4.

**Fig. 2:**
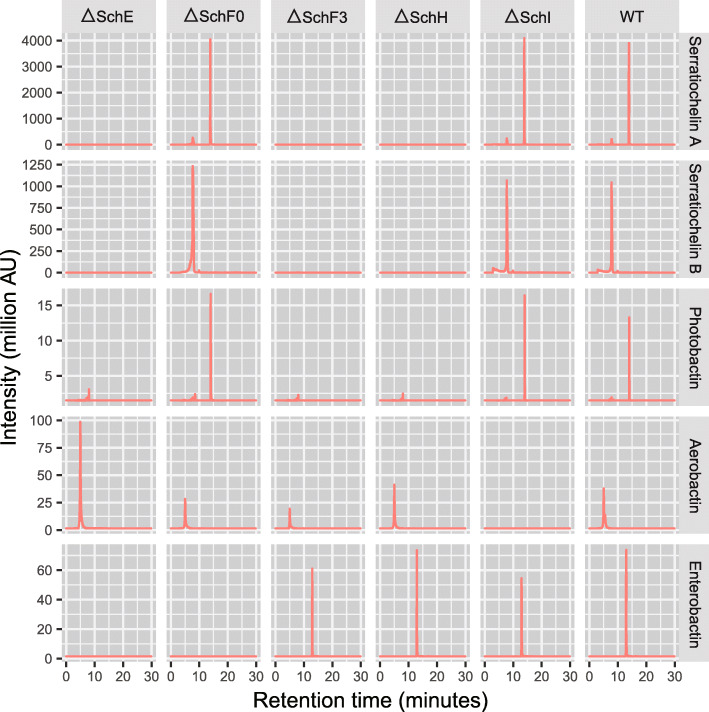
Analysis of culture supernatants from *S. plymuthica* V4 wild-type and gene deletion mutants by HPLC/MSMS. Columns: *S. plymuthica* V4 variants Δ*schE*, Δ*schF0*, Δ*schF3*, Δ*schH*, Δ*schI*, and the wild-type strain. Rows: extracted ion count in million activity units for each siderophore: serratiochelin A and B (closed and open form), photobactin, aerobactin, and enterobactin

Based on an earlier study by Seyedsayamdost et al., we were aware of the fact that gene clusters with high similarity to the *Escherichia coli* enterobactin and the *Vibrio cholera* vibriobactin producing gene clusters were present in the genome of *S. plymuthica* V4. In fact, parts of these clusters were involved in the biosynthesis of serratiochelins [16]. Seyedsayamdost et al. had confirmed by gene disruption the involvement of the enzymes SchC,-E and -B encoded by the enterobactin-like gene cluster (which we depict as cluster p1 in Fig. 1b), and SchH and -F3 encoded by the vibriobactin-like gene cluster (cluster p2 in Fig. 1b) in the biosynthesis of serratiochelins. However, the authors did not assign a function to *schF0*, one of the genes in the enterobactin-like gene cluster, which was then hypothesized to be a pseudogene [16]. Given that we had now detected enterobactin in *S. plymuthica* culture supernatants, we hypothesized that SchF0, which has high sequence similarity with EntF from *E. coli* [16], might in fact be involved in enterobactin assembly. We thus screened wild-type and SchF0 mutants of *S. plymuthica* for the production of enterobactin. We found the wild-type strain to produce enterobactin, whereas no enterobactin was produced in the mutants (Fig. 2). This suggests that *schF0* is not a pseudogene but responsible solely for enterobactin biosynthesis, while the other genes in the cluster moonlight in the biosynthesis of other siderophores. In comparison, we found that the disruption of *schF3* abolished the production of all catecholate siderophores except enterobactin (Fig. 2), in agreement with its role proposed by Seyedsayamdost et al. for the biosynthesis of serratiochelins. Our results also show that the same gene clusters, p1 and p2, are also responsible for the assembly of photobactin.

Next, we sought to investigate the role of SchE encoded in gene cluster p1 in the biosynthesis of enterobactin and photobactin. SchE had previously been seen to be essential for serratiochelin biosynthesis. Based on its homology to EntE [34], it was hypothesized to adenylate the catechol precursor 2,3-dihydroxybenzoate (DHB) and tether it to holo-SchB. This is a crucial step in catecholate siderophore assembly [34]. We screened a SchE mutant for the production of each of the aforementioned siderophores. As expected, solely aerobactin, which is a hydroxamate siderophore, was synthesized. This result agrees with our predictions and shows that no other EntE/SchE homologs are present in the genome of *S. plymuthica*.

Seyedsayamdost et al. found that in the biosynthesis of serratiochelins, the condensation of polyamines with DHB is catalyzed by *schF0* in *S. plymuthica* (encoded in cluster p2) [16]. In order to confirm the role of SchH in photobactin biosynthesis, we screened a SchH deletion mutant and found that it did not synthesize the polyamine-containing siderophores, i.e., the serratiochelins and photobactin (whose synthesis makes use of DAP and putrescine, respectively). Aerobactin was still produced, as was enterobactin. These siderophores (aerobactin and enterobactin) do not have a polyamine moiety, and so the inability to incorporate the polyamine would not have affected their production. In fact, enterobactin was overproduced by the SchH deletion mutant, in comparison to the wild-type strain (*p* = 0.004).

**Fig. 3:**
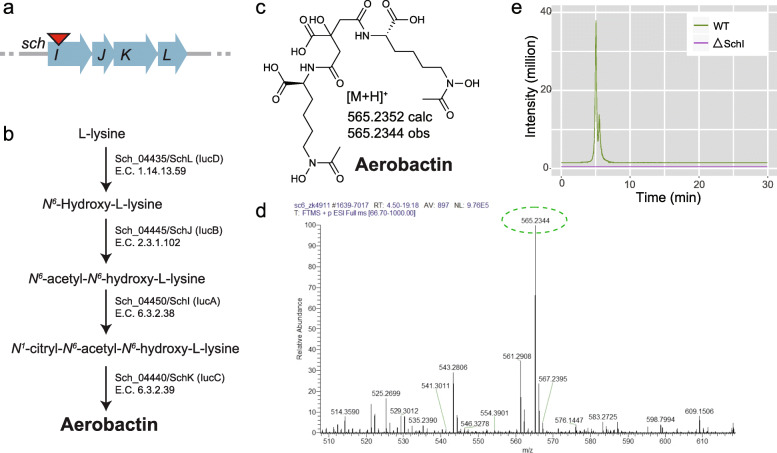
Characterization of aerobactin biosynthesis. Biosynthetic operon and respective locus of suicide vector introduction (**a**), enzymatic processes leading to aerobactin biosynthesis (**b**), calculated and observed mass of aerobactin (**c**), ESI-MS of wild-type (**d**), and aerobactin extracted ion count for the wild-type and *schI* mutant (**e**)

Lastly, given that we detected aerobactin in our samples, we decided to query the genome of *S. plymuthica* for genes homologous to those involved in the biosynthesis of aerobactin in other organisms. This enabled us to locate a chromosomal operon homologous to *iucABCD*, which we termed *schIJKL* (p3), as not to be confused with *schABCD* [33] (Fig. 3a). The four genes in this operon were highly similar to those in a *Yersinia* strain, with identities as high as 89%, as determined by pairwise analysis with the Basic Local Alignment Search Tool, BLAST2p (Additional file 1: Additional Table S1). LucA (a homolog of SchI) catalyzes the intermediate step that converts l-lysine to aerobactin [33]. To test whether this operon was indeed responsible for the production of the hydroxamate siderophore aerobactin, we built a SchI-defective mutant and tested it for the capacity to synthesize aerobactin. We found that the ΔSchI strain did not produce aerobactin (Fig. 3d, e), whereas the wild-type strain and all other mutants were capable of synthesizing aerobactin (Fig. 2). This confirms that the *schIJKL* operon (p3) is indeed responsible for the biosynthesis of aerobactin.

**Fig. 4:**
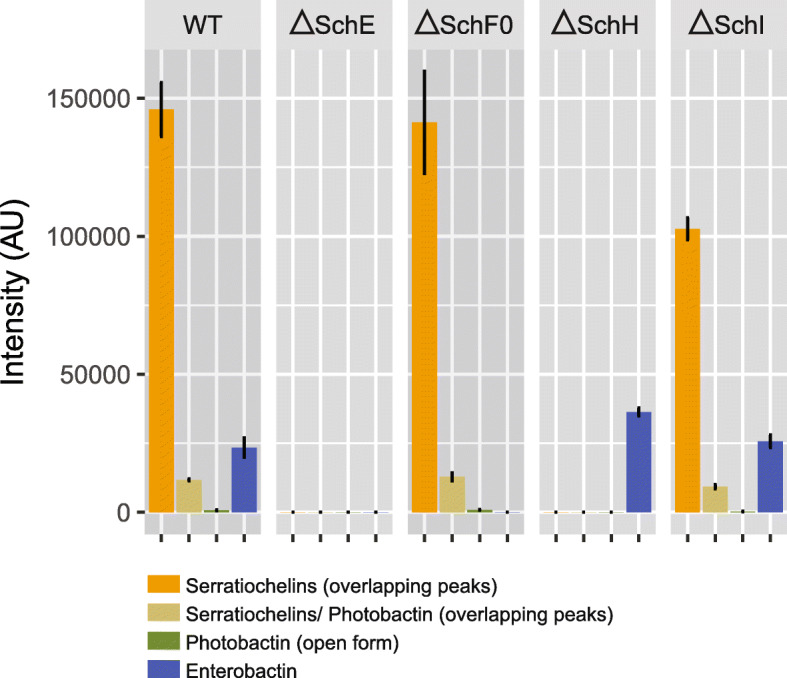
Relative abundance of serratiochelins, photobactin, and enterobactin in each mutant and wild-type strain. The low abundance of aerobactin did not allow for its relative quantification. Mean ± SD of 3 biological replicates with technical duplicates

After confirming the phenotypes caused by the knock-out of SchF0, SchE, SchH, or SchI, we quantified the relative abundances of each type of siderophore for each mutant (Fig. 4) in order to establish the potential contribution of each siderophore to the siderome, as well as its potential contribution to iron chelation, in this organism. In the wild-type strain, serratiochelin A and B represented over 80% of the siderophores and enterobactin, nearly 13%. There were small amounts of photobactin and aerobactin. In fact, the abundance of aerobactin was too low for quantification with the equipment used (Agilent single quadrupole mass spectrometer G6120a). Interestingly, knocking out SchI led to decreased production of serratiochelins (*p* = 0.004) and photobactin (*p* = 0.019). When the production of all polyamine siderophores was abolished (ΔSchH), the relative levels of enterobactin increased by ca. 50% (*p* = 0.004). The yields of all polyamine siderophores decreased when SchI was knocked out (*p* < 0.05), except for enterobactin, whose levels did not change. This suggests that the role of aerobactin in iron chelation in this organism is secondary when other siderophores are available, under the tested conditions. Moreover, our observation that enterobactin takes up the iron chelation needs arising from lack of aerobactin in the SchI knock-out strain may result from several effects. First, the polyamine siderophore biosynthesis pathway might be more tightly regulated than enterobactin biosynthesis; second, polyamine-containing siderophore biosynthesis might be more costly as it requires more enzymes and polyamines for assembly than enterobactin biosynthesis; third, enterobactin might be a more efficient siderophore with higher iron chelation efficiency than polyamine-containing siderophores. The last two possibilities would suggest that enterobactin is overall a more cost-efficient siderophore and therefore more likely to compensate for the lack of aerobactin under our experimental conditions. The relative abundances of siderophores in our experiments give a snapshot of the biosynthetic repertoire of *S. plymuthica* under our experimental conditions, but these proportions might change under different biotic and abiotic conditions, as observed in several studies with *Pseudomonas aeruginosa* and recently reviewed by Kramer et al. [35].

### Growth kinetics of *S. plymuthica* defective in the production of siderophores

**Fig. 5:**
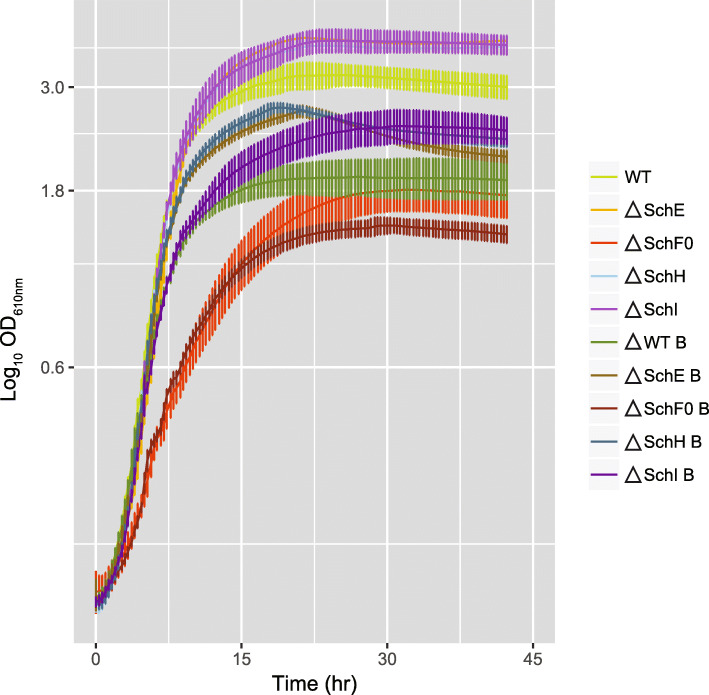
Time course of the OD_610nm_ for the mutant and wild-type strains, in the presence and absence of 0.1% bipyridyl (index B in the legend)

Given that *S. plymuthica* synthetizes a plethora of siderophores, we were interested in understanding how each of these siderophores influences bacterial growth in iron-limited conditions. Therefore, we created mutant strains defective in the production of specific types of siderophores. Then, we compared the growth of the mutants versus wild-type, in the presence or absence of bipyridyl, a soluble iron chelator. Bipyridyl chelates any soluble iron that might still be present in the minimal medium and thus leads to the activation of the siderophore-producing machinery due to low soluble iron stress. We followed the growth of the strains over time and measured their maximum growth rate and maximum OD reached (Fig. 5), in order to understand the relative importance of each group of siderophores (polyamine, catecholate, and hydroxamate siderophores). Overall, we found that the growth rate in minimal medium with bipyridyl was unchanged (Δ*schH*) or lower (wild-type and Δ*schI*) than in its absence, except for a slight increase (< 5%) for the Δ*schE* and a strong increase for the Δ*schF0* mutant (Table 1 and Additional file 1: Additional Table S6 for statistical analysis). In the case of Δ*schF0*, the maximum growth rate was, in fact, 39% (*p* = 4.38 × 10^−7^) higher in the presence of bipyridyl than in its absence, but the maximum OD_610nm_ reached was 18% lower in the presence of bipyridyl than in its absence. Compared to wild-type, we found that not producing catecholate or polyamine siderophores (SchE or SchH knockouts) increased the maximum growth rate of those mutants even in the presence of bipyridyl (Table 1). More precisely, there was an increase in growth rate of 30% for the SchH mutant and ca. 18% for the SchE mutant (*p*_SchH_ = 1.33 × 10^−35^, *p*_SchE_ = 4.43 × 10^−33^) over wild-type. Interestingly, when the organism was only incapable of producing enterobactin (SchF0 knockout), its maximum growth rate was reduced to 56% (without bipyridyl, *p* = 3.90 × 10^−21^) and 89% (with bipyridyl, *p* = 0.004) compared to wild-type. The maximum OD_610nm_ this mutant reached was also the lowest of all mutants and wild-type, even when grown in the absence of bipyridyl. To check whether these growth defects could be due to unexpected polar effects caused by the introduction of a suicide vector into *schF0*, we built a complementation strain, as well as related controls (Table 1). This complementation strain corresponds to the enterobactin-deficient strain (ΔSchF0) carrying a plasmid from which SchF0 is expressed. The complementation led the mutant strain to achieve both growth rates and ODs higher than the control (wild-type carrying an empty pTrc99A plasmid) in the presence and absence of bipyridyl (*p*_GR_ = 1.2 × 10^−3^, *p*_OD_ = 3.96 × 10^−4^, and *p*_GR_ = 6.29 × 10^−14^ and *p*_OD_ = 1.4 × 10^−3^, respectively). The higher growth rates and ODs could be due to the plasmid being present in multicopy (pBR322 ori); if this was the case, SchF0 would have been more abundant in the complementation mutant than in the wild-type. These observations indicate that the slower growth of the SchF0 knockout strain can be attributed to its inability to produce enterobactin.

**Table 1:** Maximum growth rates and OD_610nm_ of wild-type *S. plymuthica* and siderophore mutant strains grown in minimal medium, in the presence and absence of bipyridyl

Strain	No bipyridyl			0.1% bipyridyl		
	Maximum growth rate	Time (h)	Max OD_**610nm**_	Maximum growth rate	Time (h)	Max OD_**610nm**_
**WT**	0.307	25.0	3.202	0.258	27.0	1.939
**ΔSchE**	0.295	21.7	3.805	0.306	21.0	2.692
**ΔSchF0**	0.172	32.3	1.811	0.229	30.3	1.490
**ΔSchH**	0.335	23.3	3.673	0.338	19.0	2.741
**ΔSchI**	0.320	24.7	3.757	0.296	31.0	2.505
**WT pTrc**	0.114	35.0	2.761	0.118	44.7	1.874
**ΔSchF0 pTrc_F0**	0.193	30.3	3.255	0.192	34.0	2.690

Our results suggest that although *S. plymuthica* produces more serratiochelin A and B than any other siderophore under our experimental conditions, it can compensate for the loss of these siderophores (mutants Δ*schE*, Δ*schH*, and Δ*schI*) and sustain high growth rates in their absence (Table 1). In fact, the loss of enterobactin appears to have had the biggest impact on bacterial growth as seen by decreased growth rates and maximum optical densities of the enterobactin-deficient strain Δ*schF0*. Based on the increased production of enterobactin in the polyamine-containing siderophore-deficient strain Δ*schH* (Fig. 4), it can be assumed that enterobactin compensates for the loss of the other siderophores. Yet, it cannot be excluded that other undetected iron chelators play a role as well in sustaining high growth rates. We did not detect substantial amounts of aerobactin in the SchE mutant, which does not produce any of the catechol siderophores. Yet, this strain grew to a high optical density with remarkable growth rates. This ability to grow in the absence of the catechol siderophores suggests that either aerobactin is extremely efficient at mediating iron uptake, for example, through an unknown siderophore recycling mechanism, or that other iron chelators are upregulated in this strain but we have not yet detected them. Based on the improved growth of several mutants compared to wild-type, it appears that producing the spectrum of siderophores in *S. plymuthica* wild-type leads to a metabolic burden that is alleviated by inactivating some of the pathways. Another explanation is that the shift from one dominant siderophore to another triggers other physiologic responses that allows the bacteria to adopt a faster growth phenotype.

### Characterization of the polyamine production superpathway

**Fig. 6:**
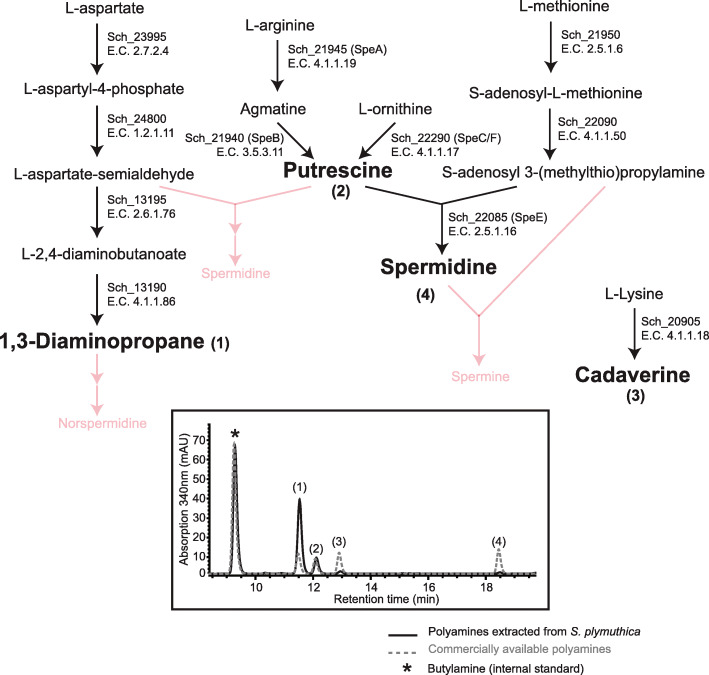
Proposed superpathway for polyamine production in *S. plymuthica* (top) and HPLC trace for the *S. plymuthica* samples and authentic standards (bottom). Polyamines not produced are displayed on the superpathway in red

The biosynthesis of the *Serratia* spp.-proprietary siderophores, the serratiochelins, is interconnected with that of polyamines. More precisely, the biosynthetic amide synthase SchH utilizes DAP as substrate for the assembly of serratiochelins [16]. We also found that this same enzyme catalyzes the condensation of putrescine (rather than DAP) with DHB, to assemble photobactin [19] (Fig. 1). The natural biosynthesis of DAP is not a generalized feature of the Enterobacteriaceae, although its heterologous expression has been achieved in *E. coli* [23, 36]. DAP is utilized in industry for the production of certain plastics [37–39] and as the basis for the production of agrochemicals [40]. Homology searches for enzymes involved in the production of polyamines in *S. plymuthica* [41] enabled us to establish a putative amine production superpathway (Fig. 6, Additional file 1: Additional Table S2). We found that this organism encoded the machinery required for the synthesis of DAP, putrescine, cadaverine, and spermidine. Spermidine could potentially be synthesized from putrescine via S-adenosylmethionine decarboxylase, which has been found to transfer the aminopropyl group from S-adenosyl-3-(methylthio) propylamine to putrescine, originating spermidine in some prokaryotes [42–44] and eukaryotes [45–47]. In some cases, spermidine can be converted to spermine by a second step that involves the transfer of an additional aminopropyl group to spermidine [48]. Given that DAP and putrescine, although not present in the growth medium, are incorporated into serratiochelins and photobactin produced by *S. plymuthica*, it can be assumed that these polyamines are synthesized endogenously.

To determine which other polyamines were produced as well, samples obtained by the lysis of cell pellets were derivatized by dansylation and analyzed by tandem mass spectrometry for the presence of DAP, cadaverine, putrescine, spermidine, spermine, N-hydroxycadaverine, and aminopropylcadaverine. Furthermore, the abundance of DAP, cadaverine, putrescine, and spermidine was assessed by UV absorbance at *λ* = 340 nm in liquid chromatography based on polyamine standards purchased from Sigma-Aldrich (Additional file 1: Additional Figs. S5- S8). *S. plymuthica* was found to produce 97.75 ± 0.01 μmol/g DW of DAP and 30.83 ± 0.003 μmol/g DW of putrescine, and small amounts of cadaverine (6.58 ± 0.01 μmol/g DW) and spermidine (2.32 ± 0.004 μmol/g DW). This level of DAP is ca. 60-fold higher than the highest reported yields of DAP naturally produced by other Proteobacteria [49].

Furthermore, part of the proposed polyamine production superpathway was confirmed by generating knockout strains and assessing their capacity to produce the predicted polyamines. The Sch_20905 knockout strain did not synthesize cadaverine (Additional file 1: Additional Fig. S9). We were unable to generate the other polyamine mutants, despite using the same approach as that used to generate all the other mutants, i.e., suicide vectors (see the “Methods” section). This suggests that DAP, putrescine, and spermidine may play essential roles in this organism. Spermidine, for example, is essential to the agrobactin-producing species *Agrobacterium tumefaciens* [50].

### Diversity of TonB-dependent receptors in *S. plymuthica*

Having elucidated the siderome of *S. plymuthica*, we were interested in understanding whether there were genes encoding the corresponding TonB-dependent receptors (TBDRs) for each type of siderophore produced, and we performed an in silico analysis. TBDRs are outer membrane proteins that, together with their inner membrane counterparts TonB, ExbB, and ExbD, transport selected siderophore-iron complexes, vitamin B12, nickel complexes, and carbohydrates into the cell [15]. To assess the diversity of TBDRs, we queried the genome of *S. plymuthica* for known genes. As expected, we found the genes encoding the putative TBDRs specific for the siderophores produced, as well as others, for a total of 12 TBDRs (Fig. 1, Additional file 1: Additional Table S3). Specifically, we identified a single putative receptor homologous to the polyamine-containing siderophore-specific receptors VuuA (vulnibactin) [51], ViuA (vibriobactin) [51], and PhuA (photobactin) [19]. Given that no other homolog was found in the genome, a single receptor may be transporting all the polyamine-containing siderophores produced by *S. plymuthica*. Additionally, *S. plymuthica* encodes a homolog of the enterobactin and colicin receptor FepA [52], a homolog of the aerobactin receptor LutA [53], two homologs of the catecholate and colicin transporter CirA [54, 55], a homolog of the catecholate and colicin transporter YiuR [56], and one homolog of IrgA, a virulence factor without known transport functions [57]. *S. plymuthica* was also found to encode receptors for fungal siderophores: one homolog of the coprogen and rhodotorulic acid transporting FhuE/PupB [58, 59], and one homolog of the ferrichrome transporting FhuA [60]. In addition, it encodes putative receptors for mammalian hemoglobin, transferrin and lactoferrin (hemlactrns receptor family); hemin (HemR/HmuR/HxuC receptors) [61–63]; and vitamin B12 and cobalamin (BtuB receptor) [64], as well as a homolog of the alcaligin, enterobactin, ferrichrome, and desferrioxamine B receptor BfrD/Fiu [65] (Fig. 1, Additional file 1: Additional Table S3). No serratiochelin-dedicated TBDR was found; thus, serratiochelin A and B may be transported into the cell by the one TBDR mentioned earlier, which has 91% sequence identity to known receptors for polyamine-containing siderophores. Our analyses confirmed that, similar to other species, the genome of *S. plymuthica* encodes TBDRs that are more diverse than the siderophores this species synthesizes. This disparity is potentially associated with siderophore piracy, by which this species obtains iron via siderophores the organism did not spend energy making [14].

### Distribution of amide synthases across bacterial orders

Having established that *S. plymuthica* produces a diversity of polyamine-containing siderophores, we were interested in determining how widespread the distribution of the amide synthases is, as this could correlate with the discovery of new polyamine-containing siderophores. Amide synthases are enzymes crucial for the assembly of siderophores that contain polyamines [16, 27]. These enzymes condense amines with other molecules, forming a carbon-nitrogen bond. To the best of our knowledge, the first amide synthase described as being involved in nonribosomal peptide assembly was VibH [27]. VibH condenses norspermidine with DHB and is involved in the assembly of vibriobactin [27]. SchH, a VibH homolog, is involved in the assembly of serratiochelins via DAP [16]. PhbG, an uncharacterized homolog of VibH and SchH, is likely the amide synthase involved in the assembly of photobactin via putrescine in *Photorhabdus asymbiotica*, though this has yet to be experimentally confirmed. We then asked how widespread the distribution of amide synthases is and whether siderophores containing polyamines have already been characterized for those same organisms.

**Fig. 7:**
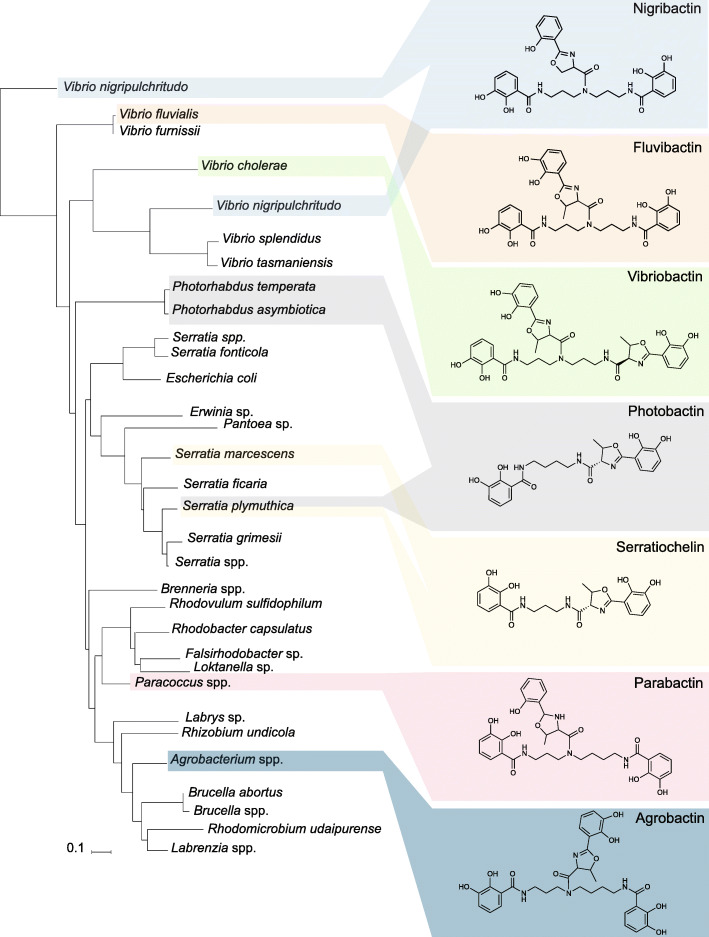
Phylogenetic tree for the distribution of amide synthases homologous to SchH across bacteria and their respective, known siderophores

A tree containing 250 SchH homologs was generated using the Distance Tree of Results tool in BLASTp (Fig. 7). Branches containing strains from the same species were collapsed for an easier interpretation of results. We then performed bibliographic searches aiming to find whether polyamine-containing siderophores had been characterized in these organisms. Of the organisms included in the tree, some have been described as producing nigribactin (*Vibrio nigripulchritudo*) [66], fluvibactin (*Vibrio fluvialis*) [67], vibriobactin (*V. cholerae*) [17], photobactin [*Photorhabdus* spp. [19] and *S. plymuthica* V4 (this study)], serratiochelin (*Serratia* spp.) [16, 68], parabactin (*Paracoccus* spp.) [69], and agrobactin (*Agrobacterium* spp.) [21]. We anticipate that many more potentially new polyamine catechol siderophores have yet to be characterized for the remaining organisms, as they encode amide synthases as well as the remaining biosynthetic machinery for the assembly of catecholate siderophores. In the particular case of *Brucella* spp., brucebactin [70], its catechol siderophore, is unstable; this instability has prevented the elucidation of its structure.

The analysis of the phylogenetic tree for SchH and its homologs did not reveal a particular evolutionary separation of the different molecules or of the aspects that make them different, such as the polyamine incorporation and condensation of DHBs on one or both primary amines (Fig. 7; Additional file 1: Additional Table S4). Amide synthases, key elements in the diversification of polyamine-containing siderophores, are encoded in diverse bacteria (Fig. 7). Looking more closely at the standalone condensation domain of these amide synthases, we sought to correlate the active site residues [27], as well as the putative donor (polyamine) and acceptor binding (carrier protein-bound DHB) residues [71], with the polyamines they condensed. For all sequences, independently of the organism and polyamine-siderophore assembled, we found that the third residue in the active site motif HH**I**XXDG was conserved, whereas it usually is not conserved in condensation domains (HHXXXDG, Additional file 1: Additional Table S4). For the donor (polyamine)-binding site, no clear sequence correlation was found. However, on the acceptor site, where DHB is presented by a carrier protein in all of the pathways, we found that the residue predicted to be crucial for substrate recognition [71] is invariably occupied by a valine (SchH residue 301). Furthermore, we identified a surface-exposed motif that appears to be characteristic of amide synthases and cannot be found in the condensation domains of other nonribosomal peptide synthetases [72]. As this motif is adjacent to W264 in VibH, a residue suggested to be important for protein-protein interaction [26], we propose that the entire motif supports the interaction with the carrier protein presenting DHB for condensation with the polyamine. To illustrate our findings, we generated a homology model for SchH based on the VibH crystal structure and mapped the described residues and motifs onto this model (Additional file 1: Additional Fig. S10).

Overall, our findings suggest that, similarly to what we observed with SchH, a single amide synthase might be capable of condensing a diversity of polyamines with the seemingly universal DHB acceptor. Nonetheless, this reaction might not be equally efficient for all polyamines, as we observed for putrescine and DAP, with the former being minimally used for siderophore assembly.

## Discussion

The capacity of pathogenic bacteria to acquire iron is intrinsically associated with their capacity to cause disease in humans [73]. Siderophores, small molecules that act as iron scavengers and transporters, have thus been repeatedly categorized as virulence factors in some of the deadliest pathogenic bacteria that humans have encountered. Both *Yersinia pestis* and *Klebsiella pneumoniae*, for example, require the siderophore yersiniabactin for colonization of a mammalian host [74]. It has been found that this same siderophore protects uropathogenic *E. coli* from copper toxicity during infection, while enterobactin allows the pathogen to survive [75]. In fact, vertebrates do not have free, soluble iron (Fe^2+^) that bacteria can use for growth. This mechanism, termed nutritional immunity, is considered a vertebrate barrier to pathogen infection [76]. Likely as a result of the pathogen-host struggle for iron, pathogens are thought to have evolved numerous ways of surviving in the host by resorting to siderophores to obtain iron. One of these strategies consists of expressing not one but a diversity of siderophores, as thoroughly studied for *P. aeruginosa* [35, 77, 78]. Similar observations have been made for non-pathogenic bacteria such as marine bacteria [79, 80] and soil bacteria [81, 82]. As recently reviewed by Kramer et al. [35] and McRose et al. [82], there are several hypotheses as to why the production of multiple siderophores might be beneficial for an organism. The most obvious ones are benefits in interspecies competition [83, 84], including the prevention of siderophore piracy [85, 86]. However, there is also strong experimental evidence that the production of multiple siderophores facilitates adaptation to changing environments (biotic and abiotic factors) and may enable biofilm formation and colonization [77, 78].

Although it rarely infects humans [87–89], *S. plymuthica* has accumulated several ways of synthesizing a diversity of siderophores and of acquiring xenosiderophores as well. In this study, we found that *S. plymuthica* diversifies its siderophore production via a natural enzymatic mixing-and-matching. Initially this organism was characterized, like other *Serratia* species, as only producing the serratiochelin siderophores [16, 68]. In this study, we detected the biosynthesis of two additional catecholate non-ribosomal siderophores—enterobactin and photobactin—as well as aerobactin, a hydroxamate siderophore. This finding is in line with recent reporting of a *Serratia* spp. strain isolated from the mosquito microbiome and found to produce several siderophores [90] as well. The presence of these additional siderophores supports the concept of the inheritance of genes as collectives [91, 92], i.e., that genes in biosynthetic gene clusters evolve together—and more quickly than their individual genes—by vertical inheritance, as well as by horizontal gene transfer. Recently, this concept has been expanded to also include the enzymatic mixing and matching events that result in the high chemical diversity of secondary metabolites observed in individual organisms [93]. The siderophores in *S. plymuthica* are a great example of chemical diversification achieved by gene cluster acquisition via horizontal gene transfer and enzymatic mixing and matching.

In fact, one of the most striking features of this organism is how efficiently it juggles two biosynthetic gene clusters to diversify the production of its catecholate siderophores. It was previously shown that the condensation domain-containing SchF0 is not involved in the assembly of serratiochelins [16]. Indeed, SchF0 is not involved in the assembly of polyamine-containing siderophores, whereas it is crucial for the assembly of enterobactin in this organism. In *S. plymuthica,* instead of SchF0, three enzymes (SchF1F2F3) assemble serratiochelins and photobactin, while the enterobactin pathway provides DHB for these siderophores. These enzymes condense the thiol-bound DHB of the aryl carrier protein SchB with the thiol-bound threonyl of SchF3, instead of the seryl of SchF0, which is used for enterobactin. Thus, the diversity of the secondary metabolome may be underestimated, in particular if prediction tools are used, and too much reliance is placed on gene clustering for functional interpretation [94].

Another level of molecular diversification occurs when SchH condenses either DAP or putrescine with the acylated dihydroxybenzoyl of SchB, and SchF3 finalizes the assembly of serratiochelins (DAP) or photobactin (putrescine). *S. plymuthica* synthesizes serratiochelins and photobactin, which implies that it can synthesize the diamines DAP and putrescine. Queries to the genome of *S. plymuthica* for all the known genes that encode the enzymes involved in the polyamine superpathways of other organisms showed that the genetic makeup required was indeed present (Additional file 1: Additional Table S2). The genetic basis of the superpathways was further confirmed by analyzing the polyamines actually produced by *S. plymuthica*, as assessed by LC-MS/MS detection and quantification of intracellular derivatized polyamine preparations (Fig. 6).

The intercommunication of enzymes encoded by genes in two independent clusters, coupled with the substrate flexibility of SchH and the polyamine production profile of this organism, allows for two pathways to generate three distinct siderophores. To the best of our knowledge, the present study is the first to report that such interplay contributes to the diversification of nonribosomal peptides in a non-engineered organism. Furthermore, aerobactin, an additional, ribosomal siderophore, is also assembled independently of the others. Nonetheless, our results indicate that the production of polyamine siderophores might be particularly costly, as knocking out their production resulted in faster-growing cells, with cultures reaching higher ODs, as well (Table 1). Despite the diversity of siderophores produced, enterobactin still seems to play a major role in this organism: under iron-limiting conditions, its absence leads to much slower growing cells that reach lower ODs. This suggests that the widespread presence of enterobactin among Proteobacteria might be due to its particularly high binding affinity for iron (K_d_ = 10^−52^ M) [95] and its cost efficiency.

The production of DAP in this organism, which is incorporated into its most abundant siderophore, was found to have the highest yield so far documented for a wild-type strain, to the best of our knowledge [23, 49, 96]. This high yield suggests that bacteria that naturally produce polyamines and incorporate them into other molecules could potentially be optimized for the industrial-level production of polyamines. Polyamines are believed to be ancient molecules; not only are they present in all domains of life but there are multiple, convergent pathways resulting in a given polyamine [46, 97]. However, it remains elusive how siderophores evolved and what selective forces gave rise to the intertwining of their biosynthetic pathways with polyamine biosynthesis. One hypothesis is that the competition for siderophores and the importance of preventing “cheaters” from utilizing xenosiderophores may be a driving force in siderophore evolution [10–12].

## Conclusions

Siderophores are molecules crucial for bacterial survival in low iron environments. Bacteria have evolved the capacity to also utilize siderophores made by other bacterial strains and to diversify their own biosynthetic repertoire for siderophores to increase their fitness. We found that *Serratia plymuthica* V4 produces five different siderophores using three gene clusters and a polyamine production superpathway. The most well-studied siderophore, enterobactin, rather than the strain’s proprietary and by far most abundant siderophore, serratiochelin, displayed a crucial role in the fitness of *S. plymuthica*. Our results also indicate that this strain is a good candidate for engineering the large-scale production of diaminopropane (DAP), as without any optimization it produced the highest amounts of DAP reported for wild-type strains.

## Materials and methods

### Strains, plasmids, and growth media

The strains and plasmids used and built for this study are listed in Table 2. Minimal medium optimized for the production of serratiochelins [16] was used for siderophore production. The minimal medium used consisted of Na2HPO4 (5.96 g/L), K2HPO4 (3.0 g/L), NH4Cl (1.0 g/L), NaCl (0.5 g/L), MgSO4 (0.058 g/L), and C6H12O6 (5.0 g/L). The final pH of the medium was 7.0.

**Table 2:** Strains and plasmids used in this study

Strain and genotype	Phenotype	Strain collection number	Reference
*S. plymuthica* V4	Wild-type	ZK4911	[16]
*S. plymuthica* V4 *schE*::GntR	Catecholate siderophore deficient	ZK4952	
*S. plymuthica* V4 *schF0*::GntR	Enterobactin deficient	ZK4962	
*S. plymuthica* V4 *schF3*::GntR	Polyamine-containing siderophore deficient	ZK4987	
*S. plymuthica* V4 *schH*::GntR	Polyamine-containing siderophore deficient	ZK4984	
*S. plymuthica* V4 *schI*::GntR	Aerobactin deficient	SA921	This study
*S. plymuthica* V4 *schF0*::GntR pTrc99A_schF0	*S. plymuthica* V4 *schF0*::GntR carrying pTrc99A_schF0	SA956	This study
*S. plymuthica* V4 *schF0*::GntR pTrc99A	*S. plymuthica* V4 *schF0*::GntR carrying pTrc99A	SA958	This study
*S. plymuthica* V4 pTRC99	*S. plymuthica* carrying pTRC99_schF0	SA960	This study
*S. plymuthica* V4 *sch_13190*::GntR	DAP defective; Sch_131390 mutant	SA976	This study
*S. plymuthica* V4 *sch_13195*::GntR	DAP defective; Sch_131395 mutant	SA977	This study
*S. plymuthica* V4 *sch_20905*::GntR	Cadaverine defective; Sch_20905 mutant	SA970	This study
*S. plymuthica* V4 *sch_21940*::GntR	Putrescine defective; Sch_21940 mutant	SA974	This study
*S. plymuthica* V4 *sch_21945*::GntR	Putrescine defective; Sch_21945 mutant	SA975	This study
*S. plymuthica* V4 *sch_22085*::GntR	Spermidine defective; Sch_22085 mutant	SA972	This study
*S. plymuthica* V4 *sch_22090*::GntR	Spermidine defective; Sch_22090 mutant	SA971	This study
*S. plymuthica* V4 *sch_22290*::GntR	Putrescine defective; Sch_22290 mutant	SA973	This study
**Plasmid**	**Genotype**	**Reference number**	**Reference**
pBTK30	R6K ori and Gen^r^ cassette	–	[98]
pSC30A	pBTK30 with a 600-bp fragment of *schI* cloned between StuI and SpeI, which replaces Mariner C9 and Amp^r^	SA918	This study
pTrc99A	IPTG-inducible expression vector, Amp^r^	–	[99]
pTrc99A_schF0	pTrc99A carrying *schF0* between NcoI and XbaI	SA956	This study

### Siderome extraction and analysis

First, we grew *S. plymuthica* in the minimal medium described above. Overnight glucose-depleted cultures of *S. plymuthica* were used to inoculate 300 mL of minimal medium containing 0.1% bipyridyl. Bacteria were grown at 250 rpm with shaking at 30 °C, until glucose depletion (monitored using QuantoFix® Glucose, Macherey-Nagel, USA). After the incubation period, the cells were spun down and the supernatant was filter-sterilized (PES, 0.22 μm) and acidified with 0.1% trifluoracetic acid (TFA, final concentration). The acidified supernatant was run through Sep-Pak tC18 (200 mg) Reversed-Phase columns (Waters®), the columns were washed with 0.1% TFA in water, and the molecules were eluted with 95% acetonitrile acidified with 0.1% TFA as well. The extracted molecules, with varying hydrophobic character, were subsequently called “secondary metabolome,” in this article.

Liquid chromatography followed by tandem mass-spectrometry (LC-MS/MS) for secondary metabolome analysis was performed at the Small Molecule Mass Spectrometry core facilities at Harvard University. Two hundred fifty-microliter aliquots of each sample were injected into an Ultimate 3000 uHPLC from Thermo Scientific equipped with a high-resolution, accurate mass Q Exactive Plus Orbitrap mass spectrometer. The elution gradient was operated over the course of 30 min at a flow rate of 0.8 mL/min, with a gradient of 10% acetonitrile (ACN) in H_2_O to 100% ACN. The detector was set to monitor positive ionization in a range from 66.7 to 1000.0 m/z (resolution 70,000 FWHM [full width at half maximum]). In order to detect and identify metabolites present in the samples, the major base peaks and their fragmentation patterns were analyzed by XCMS Online [100] and Thermo XCalibur®. The fragmentation patterns observed for the siderophores were compared with the described or predicted ones, utilizing PubChem and Chemdraw.

### Generation of siderophore knockout mutants and determination of siderophore relative abundance

All strains and plasmids used in this study are listed in Table 2; primers are listed in Additional file 1: Additional Table S5. Based on prior knowledge of serratiochelin production, we asked whether these same gene clusters were responsible for the bioassembly of enterobactin and photobactin, detected in the siderome of *S. plymuthica* [16]. We screened SchE (sch_19080), SchF0 (AHY08574.1), SchF3 (AHY05892.1), and SchH (AHY05888.1) knockout mutants for the production of each of these molecules. The mutants were kindly provided by Professor Roberto Kolter (Harvard Medical School); their construction is described elsewhere [16].

The analysis of the metabolome also revealed the presence of aerobactin, a hydroxamate siderophore [33]. In order to identify the operon responsible for the biosynthesis of aerobactin in *S. plymuthica*, we performed *iucA* homology searches in this organism using BLASTp [101, 102]. *iucA* is one of the four biosynthetic genes in the aerobactin operon, previously characterized [33]. This gene codes for a key enzyme in aerobactin biosynthesis, converting *N*^*6*^-acetyl-*N*^*6*^-hydroxy-l-lysine to *N*^1^-citryl-*N*^6^-acetyl-*N*^6^-hydroxy-l-lysine [33].

In order to search for i*ucA* in *S. plymuthica*, we generated a knockout mutant of *S. plymuthica* in which the gene *schI*, homologous to *iucA*, was disrupted by a suicide vector (pSC30A, Table 2). This suicide vector was an R6K plasmid (which replicates only in the presence of the Pir protein) carrying a 350 bp region of homology towards the 5′ end of the gene. *schI* was disrupted to disable the biosynthesis of aerobactin by *S. plymuthica*. The suicide plasmid was cloned and maintained in *E. coli* S17-λPir-1, and the plasmid was moved to *S. plymuthica* by electroporation. The R6K origin does not replicate in *S. plymuthica* but integrates at a low rate into the chromosome at the designated locus of shared homology between the plasmid and the chromosome. The transformants were plated on selective medium (10 μg/mL gentamicin), and the resulting colonies were PCR-verified for the integration of the suicide vector into *schI*.

A SchF0 knockout complementation mutant was also built, in order to confirm that the growth defects observed for the ΔSchF0 mutant resulted from the absence of SchF0 and not from polar effects. To create the complementation mutant, *schF0* was PCR-amplified from *S. plymuthica* V4 and cloned into plasmid pTRC99 by restriction digest and ligation. The construct was then electroporated into *S. plymuthica* V4 *schF0*::GntR. The empty vector pTRC99 was also electroporated into wild-type *S. plymuthica* V4, for use as control.

In order to characterize the sideromes of the wild-type strain and each of the mutants, we grew each strain in 300 mL of minimal medium supplemented with 0.1% bipyridyl, as described above. The cultures were monitored for glucose depletion and sampled at this point. Acidified (0.1% TFA) cell-free spent medium (270 mL) was supplemented with an internal control (0.5 mM Tyr-Tyr-Tyr eluent concentration, Sigma-Aldrich T2007). The spent medium was subsequently loaded into a Sep-Pak tC18 column (100 mg), as already described, and the compounds attached to the column were washed with 10% ACN (0.1% TFA) and eluted with 60%. Samples were then analyzed by high-performance liquid chromatography (HPLC) mass spectrometry (instrument: Agilent 1100, column: Agilent Zorbax Eclipse XDB-C18 80 Å, 4.6 × 150 mm, 5 μm; detector: Agilent single quadrupole mass spectrometer G6120a, injection volume 10 μL, gradient: 10% (v/v) ACN in water with 0.1% TFA for 1 min, gradient to 55% ACN with 0.1% TFA over 25 min).

### Growth dynamics of the wild-type strain and siderophore mutants

Given the diversity of siderophores produced by *S. plymuthica*, we were interested in understanding the role they played in the survival of this strain in low iron conditions. We followed their growth kinetics, utilizing a microtiter plate reader programmed to take OD_610nm_ measurements every 20 min over 42 h. Overnight (glucose depleted) cultures of the mutant and wild-type strains grown in minimal medium were used as inocula (OD_610nm_ 0.05). Cultures of each strain (200 µL) were then incubated in 96-well plates, in the presence or absence of bipyridyl (6 wells per condition and strain, experiment repeated on 3 independent occasions). The data for each strain was averaged and plotted as OD_610nm_ as a function of time (hours). This data also enable the determination of the bacterial growth rate. The standard deviation for each group of data was calculated and is represented by error bars in the plots. The programming language R was used to calculate the maximum growth rate, the time at which maximum growth occurred (package *growthrates*), and the maximum OD_610nm_.

A separate experiment was performed to compare the growth kinetics of the *schF0* complementation strain *schF0*::GntR pTRC99_schF0 and wild-type pTrc99.

### Elucidation of the polyamine biosynthesis superpathway in *S. plymuthica* and polyamine production

*S. plymuthica* synthesizes serratiochelins by incorporating DAP into the nascent molecule. We thus asked what other polyamines this organism produces and whether they are utilized to generate analogs of serratiochelin.

We started by analyzing the superpathways for polyamine production in bacteria using MetaCyc [41]. Using BLASTp [101], we queried *S. plymuthica* V4 for each of the enzymes in all three superpathways of polyamine biosynthesis. The similarity levels between the two homologous proteins were calculated using BLAST2p.

Aiming to confirm that the genes found indeed encoded enzymes involved in polyamine production, we tried to generate 8 knockout mutants using suicide vectors, as described for SchI. The genes we sought to disrupt were *sch_13190*, *sch_13195*, *sch_20905*, *sch_21940*, *sch_21945*, *sch_22085*, *sch_22090*, and *sch_22290*. Disruption of the genes *sch_21950*, *sch_23995*, and *sch_24800* was not attempted, as these genes have been deemed essential in *E. coli* [103]. After multiple attempts and even after redesigning the suicide vector to increase regions of homology of > 700 bp (in our hands, 400 bp suffice for single recombination in this strain), we were only able to disrupt *sch_20905*; therefore, we proceeded with this mutant alone.

To analyze cellular content for the polyamines predicted to be synthesized in the wild-type and mutant strains, cells were grown as described above, and upon glucose depletion 10 OD_610nm_ were pelleted. The pellets were resuspended in 500 μL of sterile water and 500 μL of 1.2 M perchloric acid (containing 1 mM butylamine as internal standard), vigorously vortexed in order to lyse the cells, and incubated for 1 h at 37 °C. The lysate was centrifuged for 20 min at 4 °C and 12,000*g*, and the supernatant was collected [104].

Dansylation of polyamines was performed as described by Smith and Davies [105] with minor modifications, as follows: to 100 μL of the supernatant above, 200 μL of saturated NaCO_3_ (130 g/L) and 400 μL of dansyl-Cl (7.5 mg/mL acetone) were added. After incubation in the dark for 1 h at 60 °C, 100 μL of proline (100 mg/mL) were added and the mixture was incubated for 30 min at 37 °C. Subsequently, the dansylated polyamines were extracted with 500 μL of toluene. For improved phase separation, the samples were centrifuged for 3 min at 3000*g*. The organic phase was dried under a nitrogen stream and the pellet was resuspended in 50 μL of methanol. For identification of the polyamines produced, a 10 μL aliquot was injected into a high-resolution, accurate mass Q Exactive Plus Orbitrap, with positive ionization and mass scan ranging from 66 to 990 m/z (resolution 17.500 FWHM), and separated over the course of 30 min at a flow rate of 0.8 mL/min, with a gradient of 10% ACN in H_2_O to 100% ACN.

Authentic standards 1,3-diaminopropane, putrescine, spermidine, spermine, and cadaverine were acquired from Sigma-Aldrich and used for determination of their fragmentation pattern, for comparison with the test samples. The standards were dansylated at the same time as the samples. The samples were analyzed as described elsewhere [105], at the Small Molecule Mass Spectrometry core facilities at Harvard University.

For quantification of the polyamines in *S. plymuthica*, samples were prepared as mentioned above except that they were resuspended in 100 μL of MeOH and analyzed by HPLC (instrument: Agilent 1100, column: Agilent Zorbax Eclipse XDB-C18 80 Å, 4.6 × 150 mm, 5 μm; detector: Agilent diode array detector G1315B, λ = 340 nm, injection volume 25 μL, gradient: 60% (v/v) MeOH in water for 1 min, gradient to 100% MeOH over 23 min). Integrated peak areas were normalized based on the internal standard and converted to concentrations in mM, based on three samples of known concentration of each authentic standard.

The dry weight was determined as the pellet biomass after 24 h at 85 °C [106] and used to calculate the concentration in mole per gram of dry weight.

### Comparative analysis of amide synthase

In order to determine the conservation and distribution of amide synthases, we queried the NCBI database for 250 homologs of SchH. In order to analyze how these homologs clustered together, based on protein sequence similarity, we downloaded the respective Newick trees (Neighbor Joining, 0.85 maximum sequence difference, Grishin distance).

The condensation domain of VibH has been thoroughly characterized by others, who revealed that its active site contains the highly conserved motif HHXXXDG [26, 72, 107]. We asked whether the three variable residues correlated with the polyamine condensed into the nascent molecule. For this, we queried the NCBI sequence database for homologs of the amide synthase SchH, from genera known to include strains that synthesize polyamine-containing siderophores. These were *S. plymuthica* (serratiochelin and photobactin), *Serratia marcescens* (serratiochelin), *Paracoccus* spp. (parabactin), *Agrobacterium*/*Rhizobacterium* (agrobactin), *Vibrio cholerae* (vibriobactin), *Vibrio fluvialis* (fluvibactin), *Vibrio nigripulchritudo* (nigribactin), and *Vibrio vulnificus* (vulnibactin). The available sequences (up to 250 per genus or strain) were aligned in NCBI (gap penalties − 11, − 1; end-gap penalties − 5, − 1; maximum cluster distance 0.8). The alignments were downloaded, further processed in CLC Sequence Viewer 7, and queried for the active site sequence. The residue variation in the conserved motif per bacterial species known to produce polyamine-containing siderophores was analyzed.

### In silico analysis of TonB-dependent siderophore receptors in *S. plymuthica* V4

Having established that *S. plymuthica* V4 produced a large repertoire of siderophores, we then asked whether this organism has a corresponding diversity of TonB-dependent siderophore receptors. In order to determine the diversity of TBDRs encoded in the chromosome of *S. plymuthica* V4, we queried it for each of the TBDR families thus far characterized (Uniprot reviewed entries only). The TBDRs found were compared to their respective protein reference sequences using NCBI’s BLAST2p tool. This tool aligns two proteins and computes their level of similarity [102].

### Homology modeling

To generate a homology model for the amide synthase SchH, we used the SWISS-MODEL server [108–110] with the VibH crystal structure as input (PDB: 1l5a, chain A; quality assessment: QMEAN − 3.2). The model was visualized using PyMol [111].

### Statistical treatment of data

Statistical significance of the results was analyzed using the unpaired, unequal variance *t* test in GraphPad Prism version 8.1. Siderophore and polyamines relative and absolute levels, respectively, were determined from 3 independent experiments, with technical duplicates. The growth curves were calculated with the data obtained from 3 independent experiments, with 6 technical replicates. The statistical significance is represented in the figures by * (*p* < 0.050), ** (*p* < 0.010), or *** (*p* < 0.001).

## Supplementary Information

**Additional file 1: Table S1.** BLAST results of *S. plymuthica* enzymes involved in aerobactin biosynthesis. **Table S2.** BLAST results of putative polyamine biosynthesis enzymes from *S. plymuthica.* **Table S3.** BLAST results of putative TonB-dependent receptors from *S. plymuthica.* **Table S4.** Amino acid sequence comparison of known amide synthases. **Table S5.** List of primers used in this study. **Table S6.** Report of statistical analysis of growth parameters or *S. plymuthica* mutant variants. **Figure S1** ESI-MS/MS of serratiochelin. **Figure S2.** ESI-MS/MS of photobactin. **Figure S3** ESI-MS/MS of enterobactin. **Figure S4.** ESI-MS/MS of aerobactin. **Figure S5.** ESI-MS/MS of dansylated 1,3-diaminopropane. **Figure S6.** ESI-MS/MS of dansylated putrescine. **Figure S7.** ESI-MS/MS of dansylated cadaverine. **Figure S8.** ESI-MS/MS of dansylated spermidine. **Figure S9.** HPLC chromatogram of dansylated polyamines extracted from *S. plymuthica*. **Figure S10.** Cartoon and surface representation of SchH modeled based on the crystal structure of VibH (PDB 1l5A).

## Data Availability

All data generated or analyzed during this study are included in this published article and its Additional File 1. The mutant variant strains generated in this study are available upon request from the corresponding author.

## Abbreviations

DAP: Diaminopropane
DHB: 2,3-Dihydroxybenzoate
TBDR: TonB-dependent receptor
OD: Optical density
WT: Wild-type
PES: Polyethersulfone
TFA: Trifluoracetic acid
ACN: Acetonitrile

## References

[1] Taylor SR (1964). Abundance of chemical elements in the continental crust: a new table. Geochim Cosmochim Acta.

[2] Andrews SC (1998). Iron storage in bacteria. Adv Microb Physiol.

[3] Andrews SC, Robinson AK, Rodriguez-Quinones F (2003). Bacterial iron homeostasis. FEMS Microbiol Rev.

[4] Ratledge C, Dover LG (2000). Iron metabolism in pathogenic bacteria. Annu Rev Microbiol.

[5] Chu BC (2010). Siderophore uptake in bacteria and the battle for iron with the host; a bird’s eye view. Biometals..

[6] Zheng TEMN (2012). Siderophore-based detection of Fe ( iii ) and microbial pathogens. Metallomics..

[7] Dunn LL, Rahmanto YS, Richardson DR (2007). Iron uptake and metabolism in the new millennium. Trends Cell Biol.

[8] Poole K, McKay GA (2016). Iron acquisition and its control in *Pseudomonas aeruginosa*: many roads lead to Rome. Front Biosci.

[9] Jurkevitch E, Hadar Y, Chen Y (1992). Differential siderophore utilization and Iron uptake by soil and rhizosphere bacteria. Appl Environ Microbiol.

[10] Cordero OX, −a. VL, DeLong EF, Polz MF. Public good dynamics drive evolution of iron acquisition strategies in natural bacterioplankton populations. Proc Natl Acad Sci 2012;109:20059–20064. 10.1073/pnas.1213344109 PMC3523850 23169633

[11] Lee W, van Baalen M, Jansen VAA (2016). Siderophore production and the evolution of investment in a public good: an adaptive dynamics approach to kin selection. J Theor Biol.

[12] Lee W, van Baalen M, Jansen VAA (2012). An evolutionary mechanism for diversity in siderophore-producing bacteria. Ecol Lett.

[13] Kümmerli R, Brown SP (2010). Molecular and regulatory properties of a public good shape the evolution of cooperation. Proc Natl Acad Sci U S A.

[14] Dumas Z, Kummerli R (2011). Cost of cooperation rules selection for cheats in bacterial metapopulations. J Evol Biol.

[15] Noinaj N, Guillier M, Barnard TJ, Buchanan SK (2010). TonB-dependent transporters: regulation, structure, and function. Annu Rev Microbiol.

[16] Seyedsayamdost MR (2012). Mixing and matching siderophore clusters: structure and biosynthesis of serratiochelins from *Serratia sp* V4. J Am Chem Soc.

[17] Griffiths GL, Sigel SP, Payne SM, Neilands JB (1984). Vibriobactin, a siderophore from *Vibrio cholerae*. J Biol Chem.

[18] Okujo N (1994). Structure of vulnibactin, a new polyamine-containing siderophore from *Vibrio vulnificus*. Biometals..

[19] Ciche TA, Blackburn M, Carney JR, Ensign JC (2003). Photobactin: a catechol siderophore produced by Photorhabdus luminescens, an entomopathogen mutually associated with Heterorhabditis bacteriophora NC1 nematodes. Society..

[20] Bergeron RJ, Dionis JB, Elliott GT, Kline SJ (1985). Mechanism and stereospecificity of the parabactin-mediated iron-transport system in *Paracoccus denitrificans*. J Biol Chem.

[21] Ong SA, Peterson T, Neilands JB (1979). Agrobactin, a siderophores from *Agrobacterium tumefaciens*. J Biol Chem.

[22] Fuell C, Elliott KA, Hanfrey CC, Franceschetti M, Michael AJ (2010). Polyamine biosynthetic diversity in plants and algae. Plant Physiol Biochem.

[23] Lee J (2009). An alternative polyamine biosynthetic pathway is widespread in bacteria and essential for biofilm formation in *Vibrio cholerae*. J Biol Chem.

[24] Michael AJ. Polyamine function in archaea and bacteria thematic minireview downloaded from. J Biol Chem. 2018;:18693. doi:10.1074/jbc.TM118.005670. 10.1074/jbc.TM118.005670 PMC6290158 30254075

[25] Pegg AE (2016). Functions of polyamines in mammals * polyamine content and metabolism.

[26] Keating TA, Marshall CG, Walsh CT, Keating AE (2002). The structure of VibH represents nonribosomal peptide synthetase condensation, cyclization and epimerization domains. Nat Struct Biol.

[27] Keating TA, Marshall CG, Walsh CT (2000). Vibriobactin biosynthesis in *Vibrio cholerae*: VibH is an amide synthase homologous to nonribosomal peptide synthetase condensation domains. Biochemistry..

[28] Rondon MR, Ballering KS, Thomas MG (2004). Identification and analysis of a siderophore biosynthetic gene cluster from *Agrobacterium tumefaciens* C58. Microbiology..

[29] Masschelein J (2013). A PKS/NRPS/FAS hybrid gene cluster from *Serratia plymuthica* RVH1 encoding the biosynthesis of three broad spectrum, zeamine-related antibiotics. PLoS One.

[30] Zhou J (2011). A novel multidomain polyketide synthase is essential for Zeamine production and the virulence of *Dickeya zeae*. Mol Plant-Microbe Interact.

[31] Pollack JR, Neilands JB (1970). Enterobactin, an iron transport compound from Salmonella typhimurium. Biochem Biophys Res Commun.

[32] O’Brien IG, Cox GB, Gibson F (1970). Biologically active compounds containing 2,3-duhydroxybenzoic acid and serine formed by *Escherichia coli*. BBA - General Subjects.

[33] De Lorenzo V, Bindereif A, Paw BH, Neilands JB (1986). Aerobactin biosynthesis and transport genes of plasmid colV-K30 in Escherichia coli K-12. J Bacteriol.

[34] Sikora AL, Wilson DJ, Aldrich CC, Blanchard JS (2010). Kinetic and inhibition studies of dihydroxybenzoate-AMP ligase from *Escherichia coli*. Biochemistry..

[35] Kramer J, Özkaya Ö, Kümmerli R (2020). Bacterial siderophores in community and host interactions. Nat Rev Microbiol.

[36] Chae TU, Kim WJ, Choi S, Park SJ, Lee SY (2015). Metabolic engineering of *Escherichia coli* for the production of 1, 3-diaminopropane, a three carbon diamine. Sci Rep.

[37] Corneillie S, Smet M (2015). Polymer chemistry PLA architectures : the role of branching. Polym Chem.

[38] Elvers B (2016). Ullmann’s polymers and plastics: products and processes.

[39] Bartkowiak M, Lewandowski G, Milchert E, Pelech R (2006). Optimization of 1, 2-diaminopropane preparation by the ammonolysis of waste. Ind Eng Chem Res.

[40] Lawrence S (2004). Amines: synthesis, properties and applications.

[41] Caspi R (2014). The MetaCyc database of metabolic pathways and enzymes and the BioCyc collection of Pathway/Genome Databases. Nucleic Acids Res.

[42] Shah P, Swiatlo E (2008). A multifaceted role for polyamines in bacterial pathogens. Mol Microbiol.

[43] Weaver RH, Herbst EJ (1957). Metabolism of diamines and polyamines in microorganisms. J Biol Chem.

[44] Stevens BL, Marcelle A (1968). Studies on the role of polyamines associated with the ribosomes from *Bacillus stearothermophilus*. J Biochem.

[45] Pegg AE (2010). Mammalian polyamine metabolism and function. Int Union Biochem Mol Biol Life J.

[46] Minguet EG, Vera-Sirera F, Marina A, Carbonell J, Blázquez MA (2008). Evolutionary diversification in polyamine biosynthesis. Mol Biol Evol.

[47] Michael AJ (2016). Polyamines in eukaryotes, bacteria, and archaea. J Biol Chem.

[48] Tabor CW, Tabor H (1985). Polyamines in microorganisms. Microbiol Rev.

[49] Busse J, Auling G (1988). Polyamine pattern as a chemotaxonomic marker within the Proteobacteria. Syst Appl Microbiol.

[50] Kim SH (2016). The essential role of spermidine in growth of *Agrobacterium tumefaciens* is determined by the 1,3-diaminopropane moiety. ACS Chem Biol.

[51] Webster A, Litwin CM (2000). Cloning and characterization of vuuA, a gene encoding the *Vibrio vulnificus* ferric vulnibactin receptor. Society..

[52] Skare JT, Bmm A, Seachord CL, Darveau RP, Postle K (1993). Energy transduction between membranes - TonB, a cytoplasmic membrane protein, can be chemically cross-linked in vivo to the outer membrane receptor FepA. J Biol Chem.

[53] Torres AG, Redford P, Welch RA, Payne SM (2001). TonB-dependent systems of uropathogenic *Escherichia coli*: Aerobactin and heme transport and TonB are required for virulence in the mouse. Infect Immun.

[54] Griggs DW, Tharp BB, Konisky J (1987). Cloning and promoter identification of the iron-regulated cir gene of *Escherichia coli*. J Bacteriol.

[55] Mcintosh MA, Earhart CF (1977). Coordinate regulation by Iron of the synthesis of phenolate compounds and three outer membrane proteins in Escherichia coli. Journal of Bacteriolo.

[56] Bosák J, Laiblová P, Smarda J, Dedicová D, Smajs D (2012). Novel colicin FY of Yersinia frederiksenii inhibits pathogenic *Yersinia* strains via YiuR-mediated reception, TonB import, and cell membrane pore formation. J Bacteriol.

[57] Goldberg MB (1992). Characterization of a Vibrio cholerae virulence factor homologous to the family of TonB-dependent proteins. Mol Microbiol.

[58] Hantke K (1983). Identification of an iron uptake system specific for coprogen and rhodotorulic acid in *Escherichia coli* K12. MGG Mol Gen Genet.

[59] Koster M, van de Vossenberg J, Leong J, Weisbeek PJ (1993). Identification and characterization of the pupB gene encoding an inducible ferric-pseudobactin receptor of *Pseudomonas putida* WCS358. Mol Microbiol.

[60] Coulton JW, Mason P, DuBow M (1983). Molecular cloning of the ferrichrome-Iron receptor of *Escherichia coli*. J Bacteriol.

[61] Cope LD, Yogev R, Muller-Eberhard U, Hansen EJ (1995). A gene cluster involved in the utilization of both free heme and heme:hemopexin by *Haemophilus influenzae* type b. J Bacteriol.

[62] Hornung JM, Jones HA, Perry RD (1996). The hmu locus of *Yersinia pestis* is essential for utilization of free haemin and haem-protein complexes as iron sources. Mol Microbiol.

[63] Stojiljkovic I, Hantke K (1992). Hemin uptake system of *Yersinia enterocolitica*: similarities with other TonB-dependent systems in gram-negative bacteria. EMBO J.

[64] Gudmondsdottir A, Bradbeer C, Kadner RJ (1988). Altered binding and transport of vitamin B12 resulting from insertion mutations in the *Escherichia coli btuB* gene. J Biol Chem.

[65] Armstrong SK, Brickman TJ, Suhadolc RJ (2012). Involvement of multiple distinct Bordetella receptor proteins in the utilization of iron liberated from transferrin by host catecholamine stress hormones. Mol Microbiol.

[66] Nielsen A, others. Nigribactin, a novel siderophore from *Vibrio nigripulchritudo*, modulates <i>Staphylococcus aureus</> virulence gene expression. Mar Drugs 2012;10:2584–2595. 10.3390/md10112584 PMC3509537 23203279

[67] Yamamoto S, others. Structures of two polyamine-containing from *Vibrio fluvialis* catecholate siderophores. J Biochem 1993;544:538–544. 10.1093/oxfordjournals.jbchem.a124079 8340347

[68] Ehlert G, Taraz K, Budzikiewicz H (1994). Serratiochelin, a new catecholate siderophore from *Serratia marcescens*. Zeitschrift fur Naturforsch - Sect C J Biosci.

[69] Peterson T, Neilands JB (1979). Revised structure of a catecholamide spermidine siderophore. From Paracoccus denitrificane. Tetrahedron Letters.

[70] González Carreró MI, Sangari FJ, Agüero J, García Lobo JM (2002). Brucella abortus strain 2308 produces brucebactin, a highly efficient catecholic siderophore. Microbiology..

[71] Bloudoff K, Alonzo DA, Schmeing TM (2016). Chemical probes allow structural insight into the condensation reaction of nonribosomal peptide Synthetases. Cell Chemical Biology.

[72] Rausch C, Hoof I, Weber T, Wohlleben W, Huson DH (2007). Phylogenetic analysis of condensation domains in NRPS sheds light on their functional evolution. BMC Evol Biol.

[73] Weinberg ED (1790). Iron availability and infection. Biochim Biophys Acta - Gen Subj.

[74] Lawlor MS, O’Connor C, Miller VL (2007). Yersiniabactin is a virulence factor for *Klebsiella pneumoniae* during pulmonary infection. Infect Immun.

[75] Chaturvedi KS, Hung CS, Crowley JR, Stapleton AE, Henderson JP (2012). The siderophore yersiniabactin binds copper to protect pathogens during infection. Nat Chem Biol.

[76] Skaar EP (2010). The battle for iron between bacterial pathogens and their vertebrate hosts. PLoS Pathog.

[77] Dumas Z, Ross-Gillespie A, Kümmerli R. Switching between apparently redundant iron-uptake mechanisms benefits bacteria in changeable environments. Proc R Soc B Biol Sci. 2013;280. 10.1098/rspb.2013.1055. 10.1098/rspb.2013.1055 PMC3712426 23760867

[78] Cornelis P, Dingemans J. *Pseudomonas aeruginosa* adapts its iron uptake strategies in function of the type of infections. Frontiers in Cellular and Infection Microbiology. 2013. doi:10.3389/fcimb.2013.00075. 10.3389/fcimb.2013.00075 PMC3827675 24294593

[79] Thode SK, Rojek E, Kozlowski M, Ahmad R, Haugen P (2018). Distribution of siderophore gene systems on a Vibrionaceae phylogeny: database searches, phylogenetic analyses and evolutionary perspectives. PLoS One.

[80] Chen J, Guo Y, Lu Y, Wang B, Sun J, Zhang H, et al. Chemistry and biology of siderophores from marine microbes. Mar Drugs. 2019;17:562. 10.3390/md17100562 PMC6836290 31569555

[81] Baars O, Zhang X, Morel FMM, Seyedsayamdost MR (2016). The siderophore metabolome of Azotobacter vinelandii. Appl Environ Microbiol.

[82] McRose DL, Seyedsayamdost MR, Morel FMM (2018). Multiple siderophores: bug or feature?. J Biol Inorg Chem.

[83] Niehus R, Picot A, Oliveira NM, Mitri S, Foster KR (2017). The evolution of siderophore production as a competitive trait. Evolution..

[84] Schiessl KT, Janssen EM-L, Kraemer SM, McNeill K, Ackermann M. Magnitude and mechanism of siderophore-mediated competition at low iron solubility in the *Pseudomonas aeruginosa* pyochelin system. Front Microbiol. 2017:1964. doi:10.3389/fmicb.2017.01964. 10.3389/fmicb.2017.01964 PMC5649157 29085345

[85] Seyedsayamdost MR, Traxler MF, Zheng S, Kolter R, Clardy J (2011). Structure and biosynthesis of amychelin, an unusual mixed-ligand siderophore from *Amycolatopsis sp* AA4. J Am Chem Soc.

[86] Challis GL, Hopwood DA (2003). Synergy and contingency as driving forces for the evolution of multiple secondary metabolite production by Streptomyces species. Proc Natl Acad Sci.

[87] Carrero P, others. Report of six cases of human infection by *Serratia plymuthica*. J Clin Microbiol 1995;33:275–276. 10.1128/jcm.33.2.275-276.1995 PMC227931 7714177

[88] Domingo D (1994). Nosocomial septicemia caused by *Serratia plymuthica*. J Clin Microbiol.

[89] Horowitz HW (1987). Serratia plymuthica sepsis associated with infection of central venous catheter. J Clin Microbiol.

[90] Ganley JG, Pandey A, Sylvester K, Lu KY, Toro-Moreno M, Rütschlin S (2020). A systematic analysis of mosquito-microbiome biosynthetic gene clusters reveals antimalarial siderophores that reduce mosquito reproduction capacity. Cell Chemical Biology.

[91] Fischbach M, Walsh C, Clardy J (2008). The evolution of gene collectives: how natural selection drives chemical innovation. Proc Natl Acad Sci U S A.

[92] Medema MH, Cimermancic P, Sali A, Takano E, Fischbach MA (2014). A systematic computational analysis of biosynthetic gene cluster evolution: lessons for engineering biosynthesis. PLoS Comput Biol.

[93] Chevrette MG, Gutiérrez-García K, Selem-Mojica N, Aguilar-Martínez C, Yañez-Olvera A, Ramos-Aboites HE (2020). Evolutionary dynamics of natural product biosynthesis in bacteria. Nat Prod Rep.

[94] Fischbach MA, Walsh CT (2006). Assembly-line enzymology for polyketide and nonribosomal peptide antibiotics: logic, machinery, and mechanisms. Chem Rev.

[95] Harris WR, others. Coordination chemistry of microbial iron transport compounds. 19. Stability constants and electrochemical behavior of ferric enterobactin and model complexes. J Am Chem Soc 1979;101:6097–6104.

[96] Hamana K, Matsuzaki S (1992). Diaminopropane occurs ubiquitously in *Acinetobacter* as the major polyamine. J Gen Appl Microbiol.

[97] Michael AJ (2016). Biosynthesis of polyamines and polyamine-containing molecules. Biochem J.

[98] Hider RC, Kong X (2010). Chemistry and biology of siderophores. Nat Prod Rep.

[99] Weiss DS, Chen JC, Ghigo JM, Boyd D, Beckwith J (1999). Localization of FtsI (PBP3) to the septal ring requires its membrane anchor, the Z ring, FtsA, FtsQ, and FtsL. J Bacteriol.

[100] Smith CA, Want EJ, Maille GO, Abagyan R, Siuzdak G (2006). XCMS: processing mass spectrometry data for metabolite profiling using nonlinear peak alignment. Matching Identification.

[101] Altschul SF, Gish W, Miller W, Myers EW, Lipman DJ (1990). Basic local alignment search tool. J Mol Biol.

[102] Altschul SF, Madden TL, Schäffer AA, Zhang J, Zhang Z, Miller W (1997). Gapped BLAST and PSI-BLAST: a new generation of protein database search programs. Nucleic Acids Res.

[103] Baba T, Ara T, Hasegawa M, Takai Y, Okumura Y, Baba M (2006). Construction of Escherichia coli K-12 in-frame, single-gene knockout mutants: the Keio collection. Mol Syst Biol.

[104] Bergeron RJ, Weimar WR (1991). Increase in spermine content coordinated with siderophore production in *Paracoccus denitrificans*. J Bacteriol.

[105] Smith MA, Davies PJ (1985). Separation and quantitation of polyamines in plant tissue by high performance liquid chromatography of their dansyl derivatives. Plant Physiol.

[106] Qian ZG, Xia XX, Lee SY (2009). Metabolic engineering of *Escherichia coli* for the production of putrescine: a four carbon diamine. Biotechnol Bioeng.

[107] Stachelhaus T, Mootz HD, Bergendahl V, a MM. Peptide bond formation in nonribosomal peptide biosynthesis. J Biol Chem 1998;273:22773–22781. 10.1074/jbc.273.35.22773 9712910

[108] Benkert P, Biasini M, Schwede T (2011). Toward the estimation of the absolute quality of individual protein structure models. Bioinformatics..

[109] Biasini M, others. SWISS-MODEL: Modelling protein tertiary and quaternary structure using evolutionary information. Nucleic Acids Res 2014;42:252–258. 10.1093/nar/gku340 PMC4086089 24782522

[110] Arnold K, Bordoli L, Kopp J, Schwede T (2006). The SWISS-MODEL workspace: a web-based environment for protein structure homology modelling. Bioinformatics..

[111] Schrödinger, LLC. The {PyMOL} Molecular Graphics System, Version~1.8. 2015.

