# Supplementary material for: Natural combinatorial genetics and prolific polyamine production enable siderophore diversification in Serratia plymuthica
Source: BMC Biol. 2021 Mar 15;19:46. doi: 10.1186/s12915-021-00971-z (PMC7962358; doi:10.1186/s12915-021-00971-z)
Supplement: Supplementary file 1 — Additional file 1: Table S1. BLAST results of S. plymuthica enzymes involved in aerobactin biosynthesis. Table S2. BLAST results of putative polyamine biosynthesis enzymes from S. plymuthica. Table S3. BLAST results of putative TonB-dependent receptors from S. plymuthica. Table S4. Amino acid sequence comparison of known amide synthases. Table S5. List of primers used in this study. Table S6. Report of statistical analysis of growth parameters or S. plymuthica mutant variants. Figure S1 ESI-MS/MS of serratiochelin. Figure S2. ESI-MS/MS of photobactin. Figure S3 ESI-MS/MS of enterobactin. Figure S4. ESI-MS/MS of aerobactin. Figure S5. ESI-MS/MS of dansylated 1,3-diaminopropane. Figure S6. ESI-MS/MS of dansylated putrescine. Figure S7. ESI-MS/MS of dansylated cadaverine. Figure S8. ESI-MS/MS of dansylated spermidine. Figure S9. HPLC chromatogram of dansylated polyamines extracted from S. plymuthica. Figure S10. Cartoon and surface representation of SchH modeled based on the crystal structure of VibH (PDB 1l5A). [file 12915_2021_971_MOESM1_ESM.docx]

**Additional file 1**

Natural combinatorial genetics and prolific polyamine production enable siderophore diversification in

*Serratia plymuthica*

Sara Cleto1,2,3, Kristina Haslinger3,4,5, Kristala L.J. Prather3,4 and Timothy K. Lu1,2,3*

1Department of Electrical Engineering and Computer Science, Massachusetts Institute of Technology

2Department of Biological Engineering, Massachusetts Institute of Technology 3Synthetic Biology Center, Massachusetts Institute of Technology 4Department of Chemical Engineering, Massachusetts Institute of Technology

5Department of Chemical and Pharmaceutical Biology, University of Groningen

* Timothy K. Lu

**Email:** [timlu@mit.edu](mailto:timlu@mit.edu)

Contents

Additional Tables 2

Additional Figures… 8

## 1

# Additional Tables

**Additional Table S1**. Enzymes involved in the biosynthesis of the hydroxamate siderophore aerobactin, identified by their protein name and locS*plymuthica* V4, and compared to enzymes from other aerobactin- producing bacteria on amino acid level.

| **Protein** | **Comparison** | **Positive s** | **E-**  **value** | **Identit y** | **Gaps** |
| --- | --- | --- | --- | --- | --- |
| IucD/SchL (sch_04435) | *Y. pekkanenii*  WP_049612679.1 | 93% | 0.0 | 89% | 0% (n=0) |
|  | *E. coli*  KRT38817.1 | 80% | 0.0 | 70% | 0% (n=0) |
|  | *E. aerogenes*  CZY40742.1 | 81% | 0.0 | 71% | 0% (n=0) |
| IucC/SchK (sch_04440) | *Y. pekkanenii*  WP_049612681.1 | 91% | 0.0 | 87% | 0% (n=0) |
|  | *E. coli*  KRT38816.1 | 84% | 0.0 | 72% | 0% (n=2) |
|  | *E. aerogenes*  SAC04707.1 | 83% | 5.0 x10-166 | 72% | 0% (n=2) |
| IucB/SchJ (sch_04445) | *Y. enterocolitica*  WP_050143497.1 | 91% | 0.0 | 85% | 0% (n=0) |
|  | *E. coli*  KRT38815.1 | 80% | 5.0 x10-163 | 69% | 0% (n=0) |
|  | *E. aerogenes*  SAC04673.1 | 81% | 5.0 x10-166 | 70% | 0% (n=0) |
| IucA/SchI (sch_04450) | *Y. enterocolitica*  WP_050143497.1 | 91% | 0.0 | 85% | 0% (n=0) |
|  | *E. coli*  KRT38814.1 | 82% | 0.0 | 73% | 0% (n=0) |
|  | *E. aerogenes*  SAC17751.1 | 83% | 0.0 | 73% | 0% (n=0) |

**Additional Table S2**. *S. plymuthica* proteins involved in the conversion of specific amino acids to polyamines and comparison to reference sequences on amino acid level.

| **Enzyme activity** | **Protein** | **Reference sequence** | **Max score** | **Total score** | **E-**  **value** | **Identity** |
| --- | --- | --- | --- | --- | --- | --- |
| Aspartate kinase | Sch_23995 | WP_015962105.1 | 796 | 796 | 0.0 | 89% |
| Aspartate-semialdehyde dehydrogenase | Sch_24800 | WP_009111079.1 | 696 | 696 | 0.0 | 91% |
| Diaminobutyrate-2- oxoglutarate aminotransferase | Sch_13195 | WP_017802287.1 | 894 | 894 | 0.0 | 94% |
| 2,4-diaminobutyrate decarboxylase | Sch_13190 | WP_014543183.1 | 933 | 933 | 0.0 | 92% |
| Arginine decarboxylase | Sch_21945 | WP_019082398.1 | 1240 | 1240 | 0.0 | 91% |
| S-adenosylmethionine synthetase | Sch_21950 | WP_005123306.1 | 764 | 764 | 0.0 | 95% |
| Agmatinase | Sch_21940 | WP_008499737.1 | 566 | 566 | 0.0 | 88% |
| Ornithine decarboxylase | Sch_22290 | WP_006327912.1 | 1500 | 1500 | 0.0 | 99% |
| Spermidine synthase | Sch_22085 | Q66EH3.1 | 552 | 552 | 0.0 | 91% |
| S-adenosylmethionine decarboxylase | Sch_22090 | Q66EH4.1 | 509 | 509 | 0.0 | 91% |
| Lysine decarboxylase | Sch_20905 | WP_013814260.1 | 1489 | 1489 | 0.0 | 99% |

**Additional Table S3.** Putative TonB-dependent receptor homologs found in the chromosome of *S. plymuthica*, molecule transported, and level of similarity with the reference sequence.

| **TonB-dependent receptors** | **Putative homolog** | **Homolog reference sequence** | **Molecule transported** | **Max scor e** | **Total scor e** | **Identit y** |
| --- | --- | --- | --- | --- | --- | --- |
| FhuE/PupB/FptA/FpvA/PbuA/Pup A | Sch_1944 5 | WP_012146208.  1 | Coprogen, rhodotorulic acid, pseudobactin | 1375 | 1375 | 91% |
| FhuA/FatA/Fct | Sch_2198 0 | WP_004947851.  1 | Ferrichrome, ferrichrysobactin, photobactin | 1509 | 1509 | 99% |
| Fep/Cbt/ Cbr/FeuB/FepA/PfeA/IroN/BfeA | Sch_1913 0 | WP_006327062.  1 | Enterobactin; colicins B, D | 1562 | 1562 | 99% |
| BfrD/Fiu/FoxA | Sch_1481 5 | WP_004706957.  1 | Ferrioxamine B | 1057 | 1057 | 70% |
| Cir/IrgA/YiuR | Sch_1800 5 | WP_016926997.  1 | Catecholate, colicin | 1129 | 1129 | 83% |
| CirA/YncD | Sch_1572 5 | WP_006321367.  1 | Catecholate, colicin | 1447 | 1447 | 98% |
|  | Sch_0280 0 | WP_006323436.  1 |  | 1533 | 1533 | 100% |
| VuuA/ViuA/PhuA | Sch_0472 5 | WP_017891746.  1 | Vulnibactin, vibriobactin, photobactin, serratiochelin? | 1377 | 1377 | 94% |
| IutA/RhtA | Sch_0443 0 | WP_002211646.  1 | Aerobactin/rhizobacti n | 1327 | 1327 | 87% |
| Hemlactrns | Sch_1299 5 | WP_006318362.  1 | Hemoglobin, transferrin, lactoferrin | 1575 | 1575 | 97% |
| HemR/HmuR/HxuC | Sch_1174 5 | WP_011815406.  1 | Hemin | 1036 | 1036 | 70% |
| BtuB | Sch_2540 0 |  | Vitamin B12/cobalamin |  |  |  |

Maximum score = Total score E-value 0.0 for all sequences

**Additional Table S4.** Amide synthase active site residues by species and polyamine condensed, based on NCBI sequence data and characterized siderophores.

| **Species (number of sequences)** | **Variable residues in active site motif (% of sequences)** | **Proposed acceptor binding motif**  **(% of sequences)** | **Polyamine** |
| --- | --- | --- | --- |
| *S. plymuthica*  (n=9) | III (100%) | MNRRSS (100%) | Diaminopropane/putrescine |
| *S. marcescens*  (n=64) | ILV (86%) ILI (14%) | MNRRGQ (77%)  LNRRGQ (20%)  LNRRGH (3%) | Diaminopropane |
| *Photorhabdus* spp. (n=9) | ILL (89%) IIL (11%) | MNRRTR (100%) | Putrescine |
| *Agrobacterium/Rhizobacterium*  (n=32) | IVV (97%) IIV (3%) | MNRRSS (100%) | Spermidine |
| *Paracoccus* spp. (n=9) | IAL (100%) | MSRMGS (88.9%)  LSRMGS (11.1%) |  |
| *Vibrio cholerae*  (n=90) | IVL (98%) IIL (2%) | MNRWGS (100%) | Norspermidine |
| *Vibrio fluvialis*  (n=13) | IVM (100%) | MSRWGS (100%) |  |
| *Vibrio nigripulchritudo*  (n=11) | IAV (64%) IVL (36%) | MNRFGS (37%)  MNRMGN (63%) |  |
| *Vibrio vulnificus*  (n=42) | IAL (100%) | LNRMGN (100%) |  |

**Additional Table S5.** List of primers used in this study to generate the plasmids listed in Table 2. Reverse 1 and 2 represent the reverse primers used for the first and second size homology for gene disruption, respectively.

| **Gene** | **Primer sequence (5'-3')** | | |
| --- | --- | --- | --- |
|  | **Forward** | **Reverse 1** | **Reverse 2** |
| **Deletions** | | | |
| *sch_04450 (schI)* | GCGTGAAACCCAGGACT GG | CGCTTATCGACGGCAAA CCAG | GGTAAAGTGCGCCAACAG CTG |
| *sch_13190* | AGTAGCCAGGCCGTTGT GCAG | CTGGTGAACACCCCGG CATCG | CCGGCCTGAACTTCATCG ATG |
| *sch_13195* | AGTTGCGAATTCATTAAA CGG | CCGCCAGCGCACCATG G | GTGTTTCGCCGCCAACTG C |
| *sch_20905* | GTCTATTACAAAGATGAG CCTATTCG | CTGGAAGGCAGTGCCG GC | GCCATTTGCCGGAGCCCT G |
| *sch_21940* | AGTCCGATAATTCCTTAG TGTCC | GCGGCAGCGTGACGAA GTG |  |
| *sch_21945* | CAGGAGGTTGCCATGAA TGACCG | GCCATTTGCCGGAGCC CTG | CAATCAGGCCTTCGTTCG GC |
| *sch_22085* | AGTACTTCTCGGTAGAG AATGTGCTG | TCGTCGTAGGAGCCGG C | GTGCCATCGTAAGTACCG AGC |
| *sch_22090* | GGCTTCAACAACCTGAC GAAGAGC | TGCACAATCCGCCTTCT GG | GCTGTTGACCGCTTCGTC C |
| *sch_22290* | GCTGCGCCAGATTGTCC G | GCCTTCGTGGATCAGCA GG | GACGTCCATCATGTGGTA CAGCG |
| *sch_13190* | AGTAGCCAGGCCGTTGT GCAG | CTGGTGAACACCCCGG CATCG | CCGGCCTGAACTTCATCG ATG |
| *sch_13195* | AGTTGCGAATTCATTAAA CGG | CCGCCAGCGCACCATG G | GTGTTTCGCCGCCAACTG C |
| *sch_20905* | GTCTATTACAAAGATGAG CCTATTCG | CTGGAAGGCAGTGCCG GC | GCCATTTGCCGGAGCCCT G |
| **Complementation** | | | |
| *schF0* | ATGGCGCCATTACCGTT AACAGG | TTACAGCGCTCTTAGAA TGCGG |  |

**Additional Table S6.** Comparison of growth parameters of various *S. plymuthica* strains investigated in this study including results of Welsh’s t-test.

| group 2 | Δ*schF0* with bipyridyl | Δ*schF0* with bipyridyl | Δ*schH* with bipyridyl | Δ*schE* with bipyridyl | Δ*schF0* | Δ*schF0* with bipyridyl | Δ*schF0* pTrc_F0 | | Δ*schF0* pTrc_F0 with bipyridyl | |
| --- | --- | --- | --- | --- | --- | --- | --- | --- | --- | --- |
| group 1 | Δ*schF0* | Δ*schF0* | WT with bipyridyl | WT with bipyridyl | WT | WT with bipyridyl | WT pTrc | | WT pTrc with bipyridyl | |
| growth parameter | µmax | OD600max | µmax | µmax | µmax | µmax | µmax | OD600max | µmax | OD600max |
| Unpaired t test with Welch's correction | | | | | | | | | | |
| P value | <0.0001 | <0.0001 | <0.0001 | <0.0001 | <0.0001 | 0.0039 | 0.0012 | <0.0001 | <0.0001 | 0.0014 |
| P value | **** | **** | **** | **** | **** | ** | ** | **** | **** | ** |
| summary |  |  |  |  |  |  |  |  |  |  |
| One- or two- | Two-tailed | Two-tailed | Two-tailed | Two-tailed | Two-tailed | Two-tailed | Two-tailed | Two-tailed | Two-tailed | Two-tailed |
| tailed P value? |  |  |  |  |  |  |  |  |  |  |
| Welch-corrected | t=6.226, | t=6.388, | t=10.13, | t=7.377, | t=21.13, | t=3.110, | t=4.054, | t=14.00, | t=7.164, | t=4.201, |
| t, df | df=31.23 | df=20.79 | df=33.25 | df=23.23 | df=28.45 | df=32.55 | df=13.66 | df=17.08 | df=11.95 | df=11.22 |
| F test to compare variances | | | | | | | | | | |
| F, DFn, Dfd | 1.848, 17, | 8.856, 17, | 1.354, 17, | 5.268, 17, | 2.581, 17, | 1.534, 17, | 8.159, 11, | 3.319, 11, | 23.23, 11, | 99.40, 11, |
|  | 17 | 17 | 17 | 17 | 17 | 17 | 11 | 11 | 11 | 11 |
| P value | 0.2158 | <0.0001 | 0.539 | 0.0013 | 0.0584 | 0.3864 | 0.0016 | 0.0585 | <0.0001 | <0.0001 |
| P value | ns | **** | ns | ** | ns | ns | ** | * | ** | **** |
| summary |  |  |  |  |  |  |  |  |  |  |
| N group 1 | 18  18 | 18  18 | 18  18 | 18  18 | 18  18 | 18  18 | 12  12 | 12  12 | 12  12 | 12  12 |
| group 2 |  |  |  |  |  |  |  |  |  |  |

## 7

Additional Figures


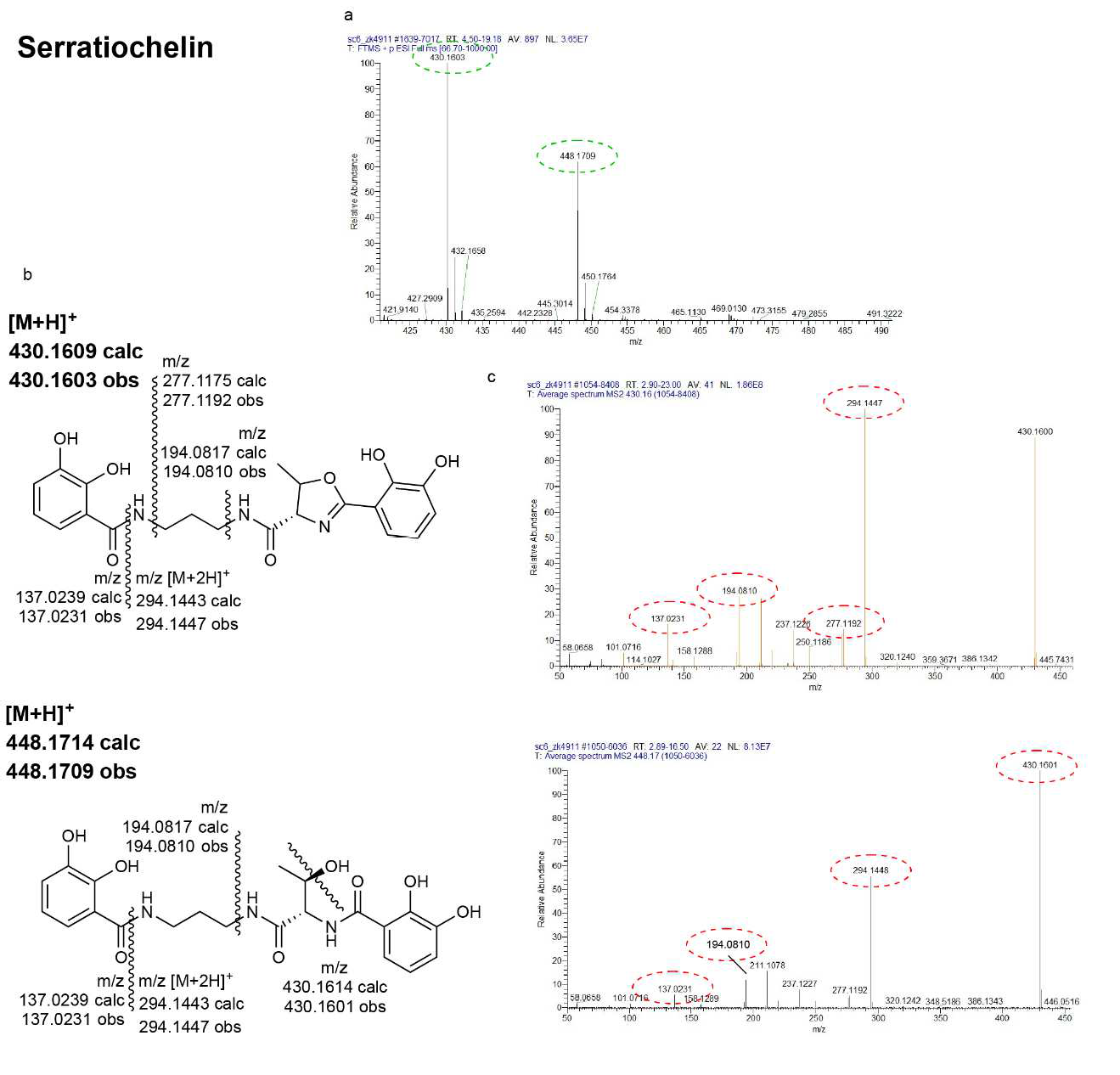


**Serratiochelin A**

**Serratiochelin B**

**Additional Figure S1:** ESI-MS for open and closed-ring serratiochelin (a). Chemical structures, calculated and observed masses (b) and observed ESI-MS/MS (c). The peak circled in green corresponds to the expected masses of the unfragmented molecules and the peaks circled in red correspond to the fragmentation products.

## 8


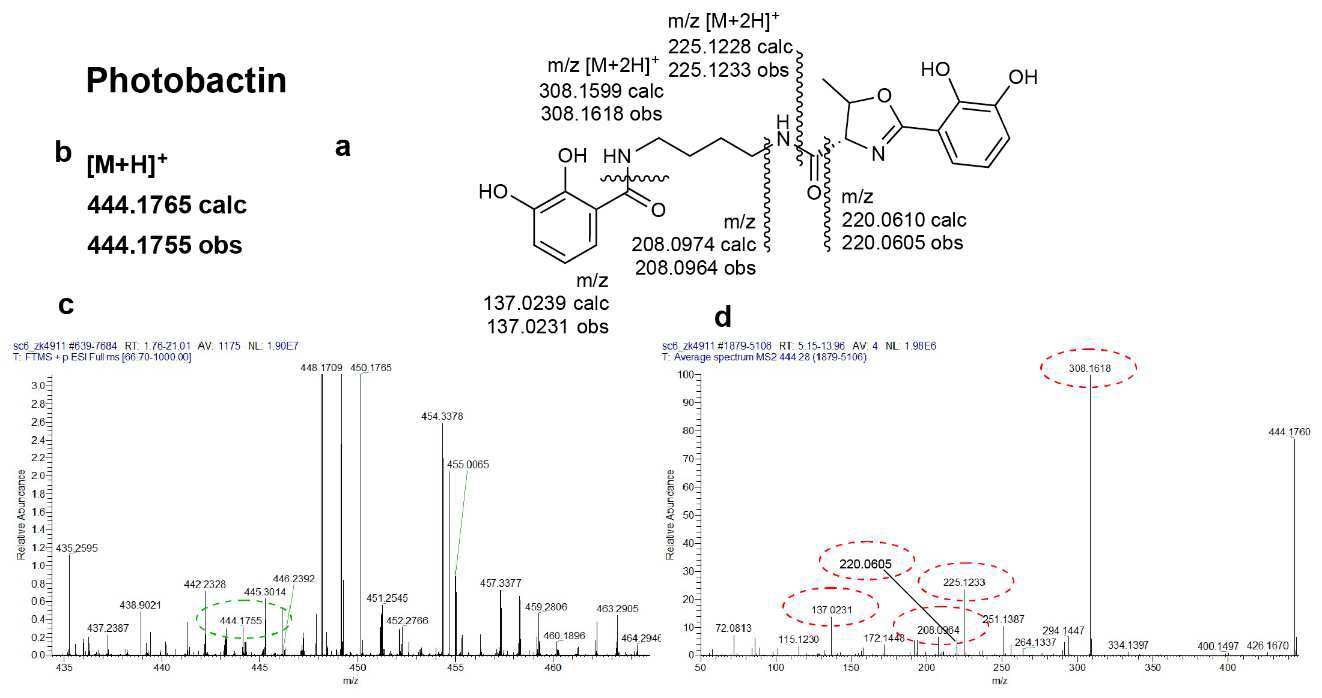


**Additional Figure S2:** ESI-MS/MS of photobactin. Chemical structure including suggested fragmentation (a), calculated and observed mass (b), observed ESI-MS (c) and observed ESI-MS/MS (d). The peak circled in green corresponds to the expected mass of the unfragmented molecules and the peaks circled in red correspond to the fragmentation products.


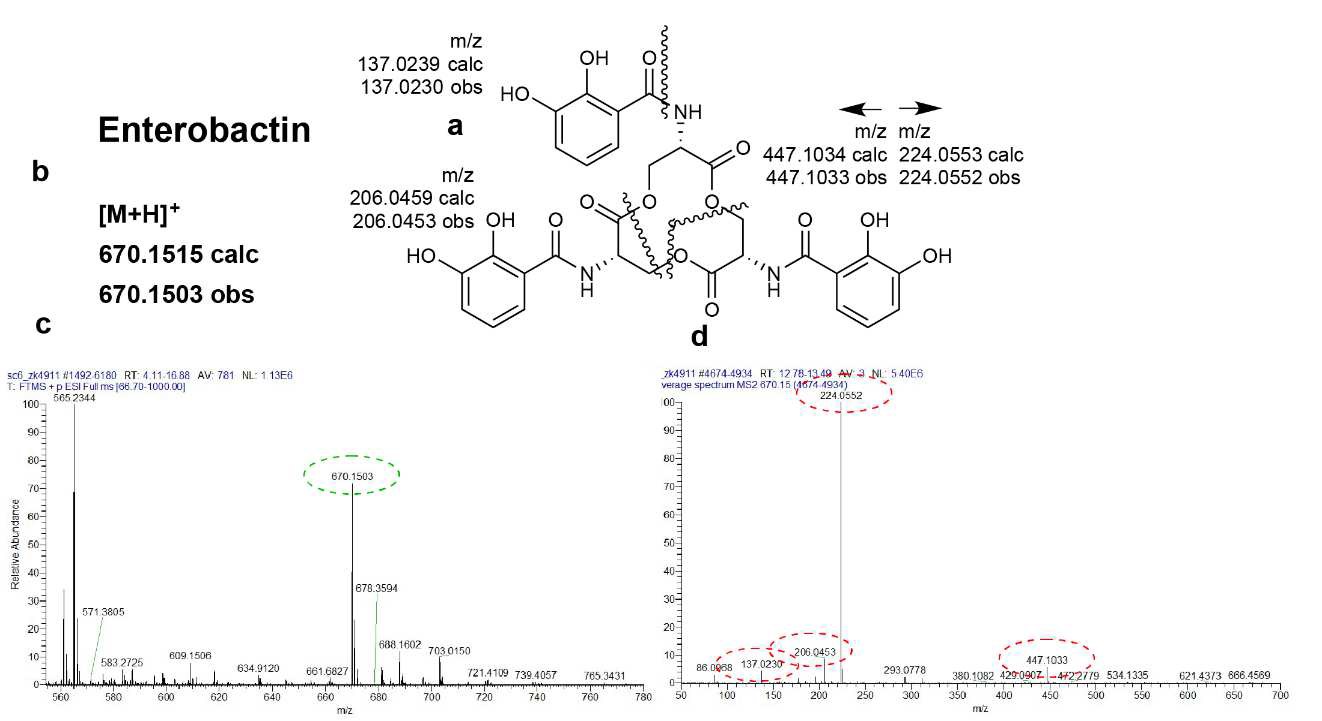


**Additional Figure S3:** ESI-MS/MS of enterobactin. Chemical structure including suggested fragmentation (a), calculated and observed mass (b), observed ESI-MS (c) and observed ESI-MS/MS (d). The peak circled in green corresponds to the expected mass of the unfragmented molecules and the peaks circled in red correspond to the fragmentation products.


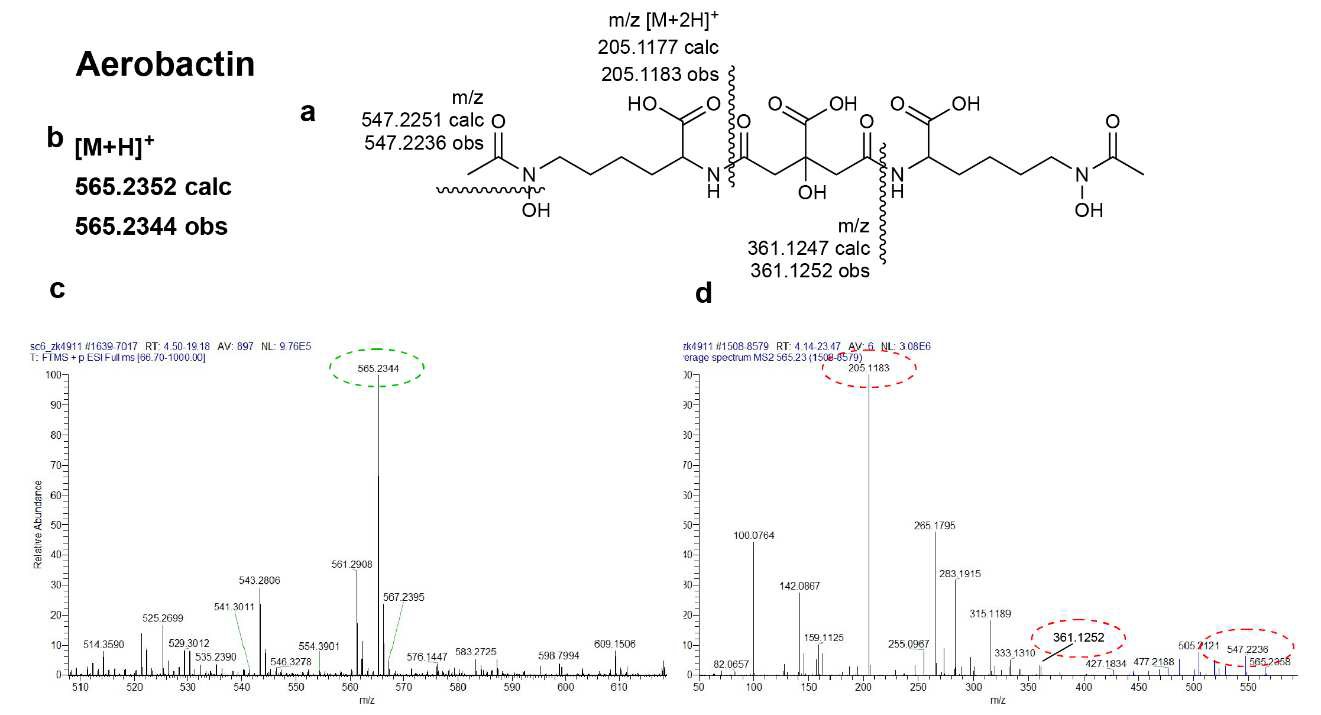


**Additional Figure S4:** ESI-MS/MS of aerobactin. Chemical structure including suggested fragmentation (a), calculated and observed mass (b), observed ESI-MS (c) and observed ESI-MS/MS (d). The peak circled in green corresponds to the expected mass of the unfragmented molecules and the peaks circled in red correspond to the fragmentation products.


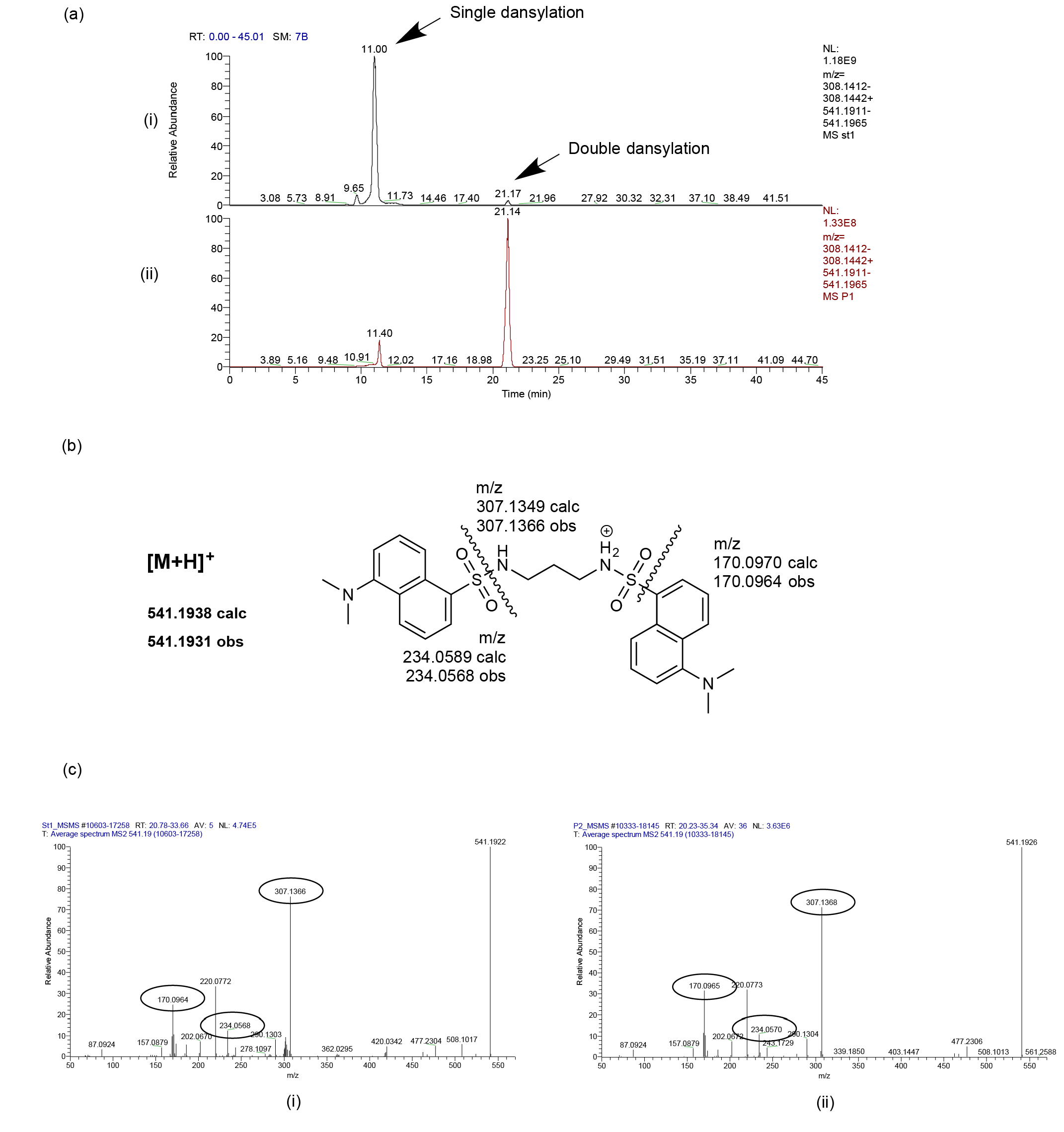


**Additional Figure S5:** Analysis of dansylated 1,3- Diaminopropane: extracted ion count for partially (i) and fully (ii) dansylated polyamines (a); structure, exact masses and fragmentation patterns (b); ESI-MS/MS fragmentation patterns for (i) and (ii) (c). Circled peaks correspond to expected fragments.


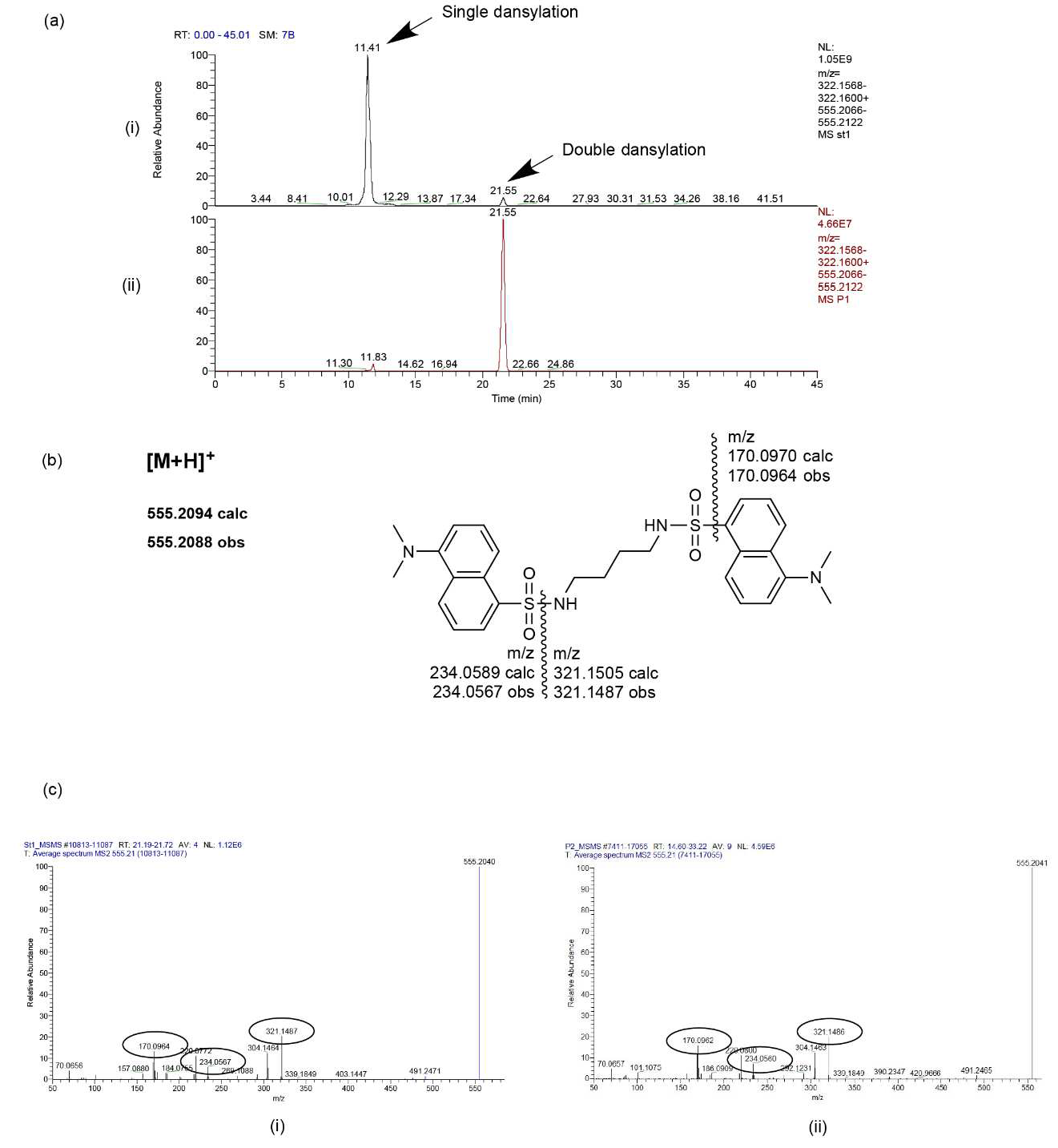


**Additional Figure S6:** Analysis of dansylated putrescine: extracted ion count for partially (i) and fully (ii) dansylated polyamines (a); structure, exact masses and fragmentation patterns (b); ESI-MS/MS fragmentation patterns for (i) and

(ii) (c). Circled peaks correspond to expected fragments.


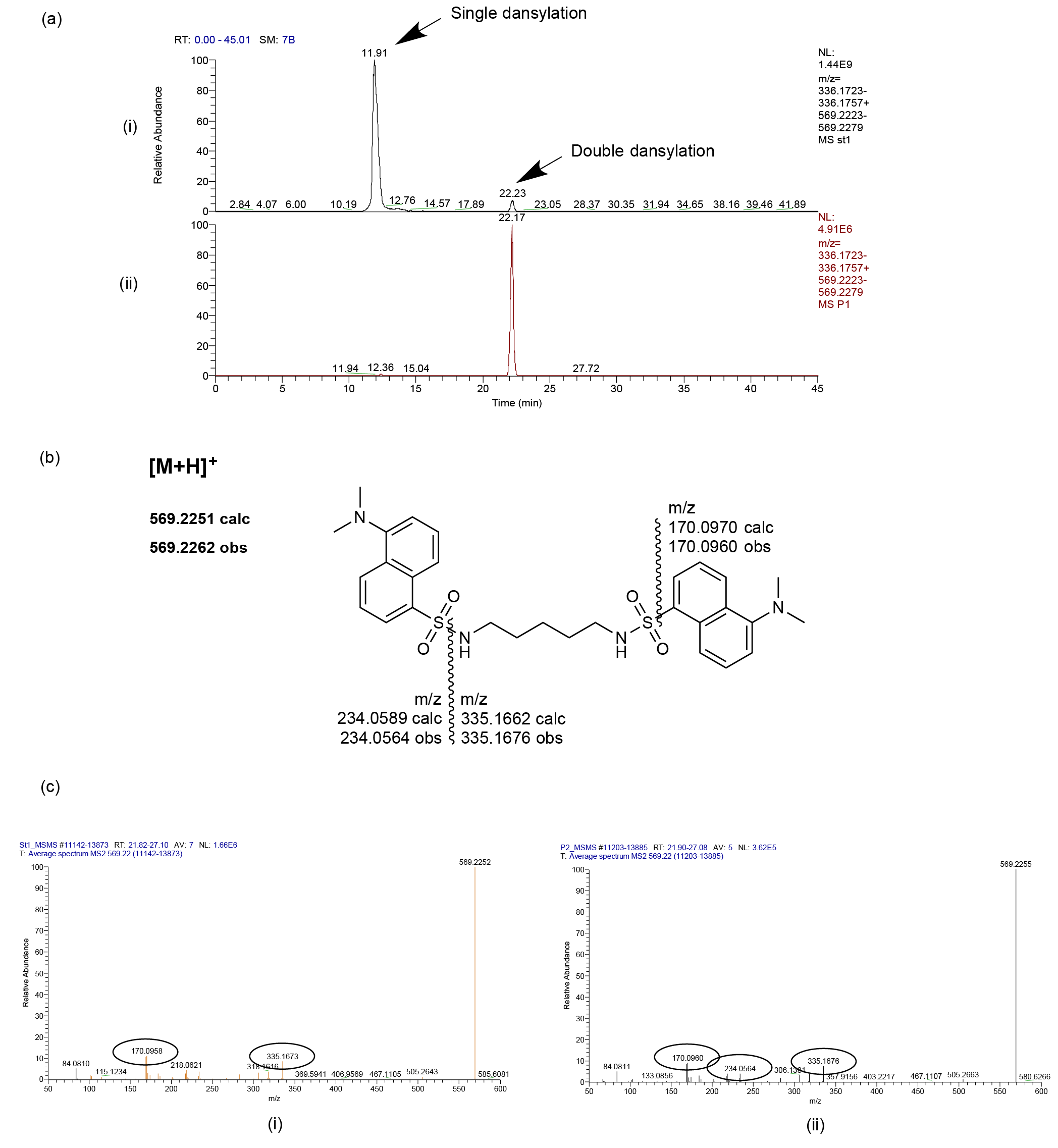


**Additional Figure S7:** Analysis of dansylated cadaverine: extracted ion count for partially (i) and fully (ii) dansylated polyamines (a); structure, exact masses and fragmentation patterns (b); ESI-MS/MS fragmentation patterns for (i) and

(ii) (c). Circled peaks correspond to expected fragments.


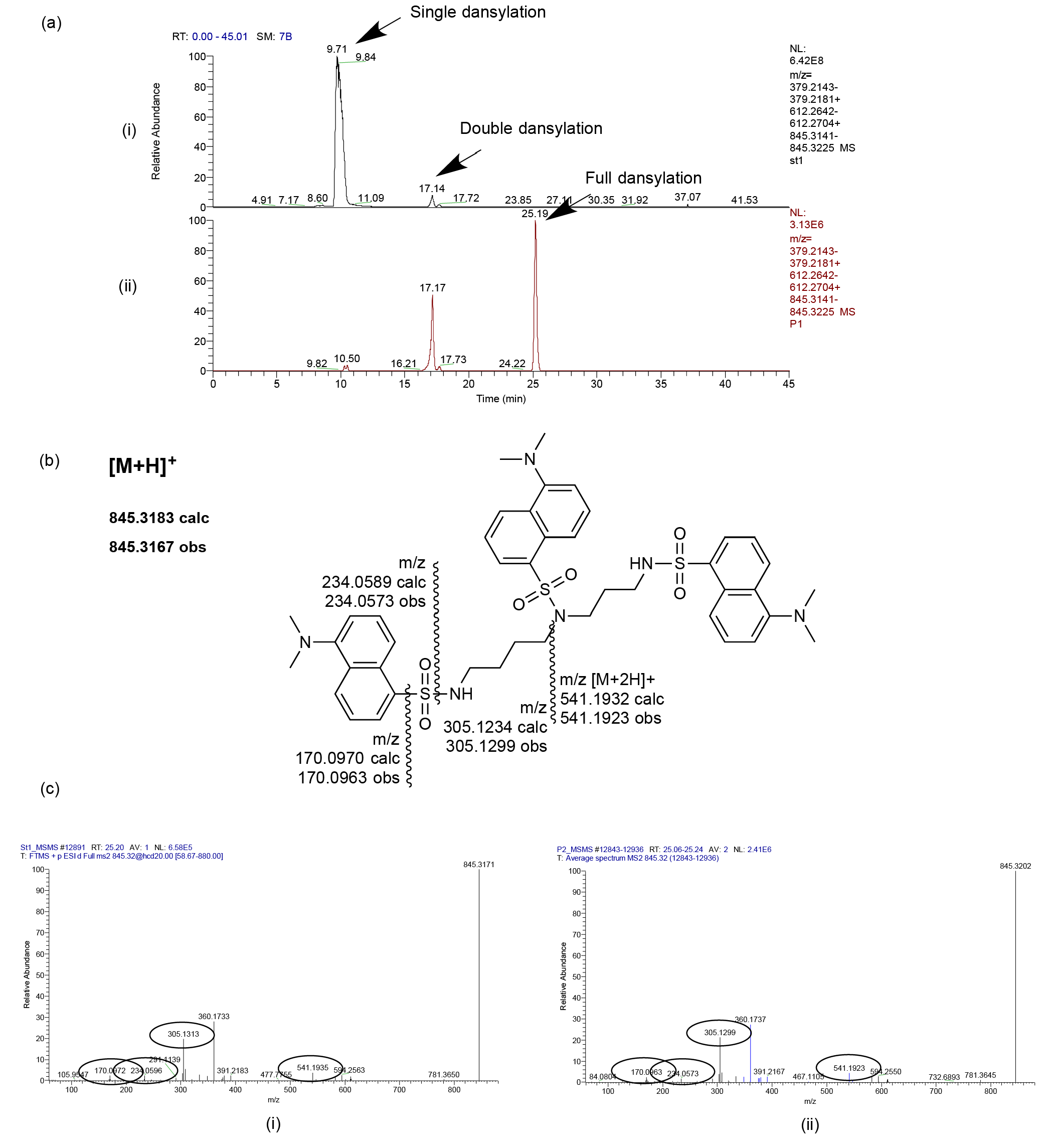


**Additional Figure S8:** Analysis of dansylated spermidine: extracted ion count for partially (i) and fully (ii) dansylated polyamines (a); structure, exact masses and fragmentation patterns (b); ESI-MS/MS fragmentation patterns for (i) and

(ii) (c). Circled peaks correspond to expected fragments.


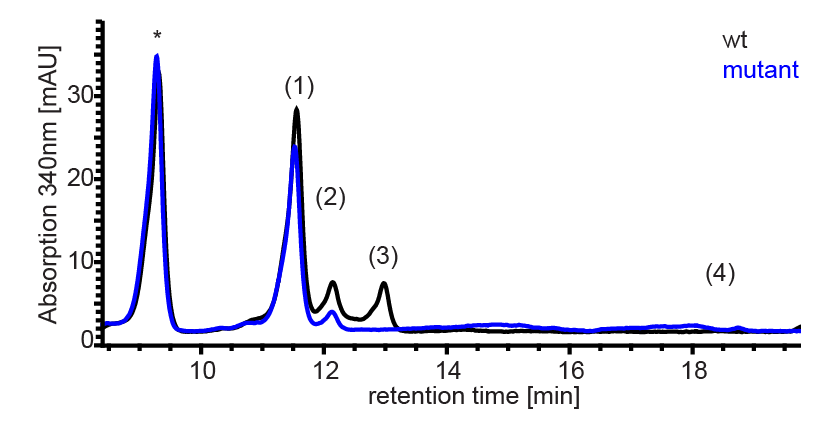


**Additional Figure S9.** HPLC chromatogram of dansylated polyamines extracted from *S. plymuthica* wildtype (black) and Sch_20905 knock-out strain (blue). Peak assignments based on data shown in Figure 6: * butylamine (internal standard), (1) 1,3-diaminopropane, (2) putrescine, (3) cadaverine, (4) spermidine.


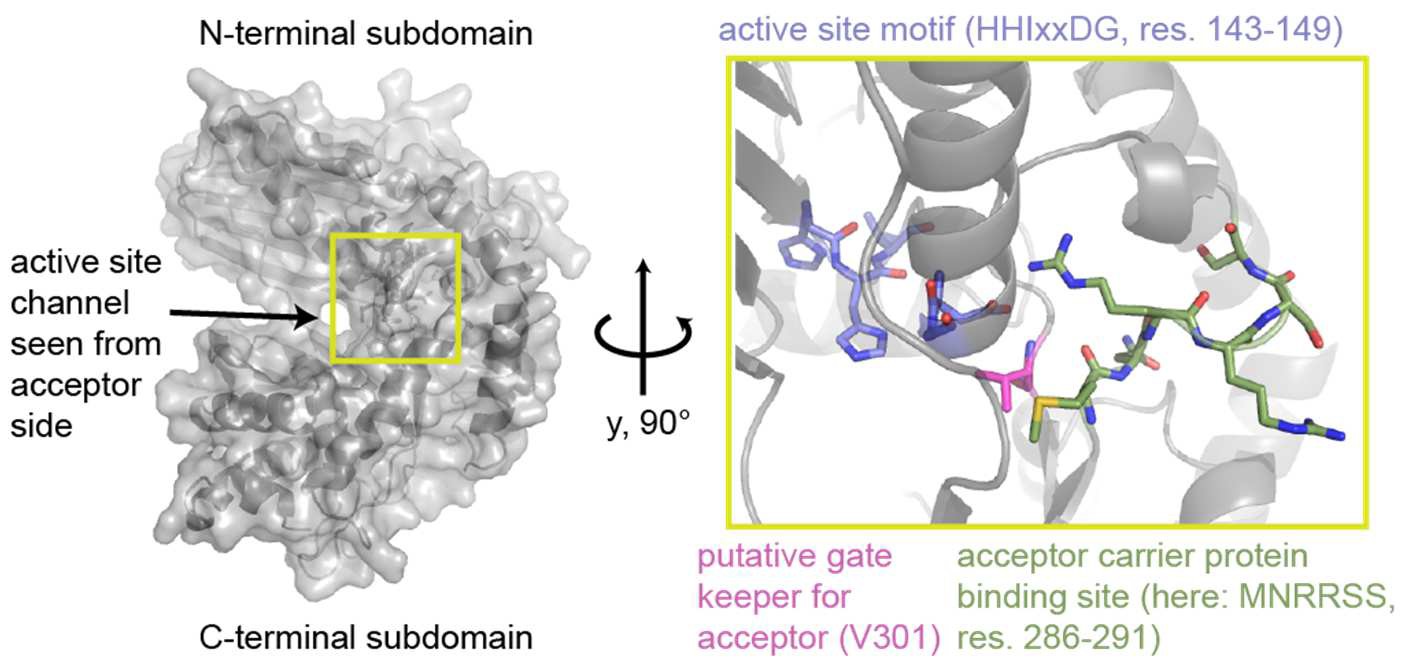


**Additional Figure S10.** Cartoon and surface representation of SchH modeled based on the crystal structure of VibH (PDB 1l5A). Area in yellow box shows the active site channel with residues forming the active site motif in blue sticks, acceptor carrier protein binding site in green sticks, and the putative gate keeper residue V301 in pink sticks (dark blue=nitrogen atoms, red=oxygen atoms, yellow=sulfur atoms).
